# The relationship between nutritional status at the time of stroke on adverse outcomes: a systematic review and meta-analysis of prospective cohort studies

**DOI:** 10.1093/nutrit/nuac034

**Published:** 2022-05-27

**Authors:** Arnav Mehta, Lorenzo De Paola, Tiberiu A Pana, Ben Carter, Roy L Soiza, Mohannad W Kafri, John F Potter, Mamas A Mamas, Phyo K Myint

**Affiliations:** Aberdeen Cardiovascular & Diabetes Centre, School of Medicine, Medical Sciences & Nutrition, University of Aberdeen, Aberdeen, Scotland, United Kingdom; Ageing Clinical and Experimental Research Team, Institute of Applied Health Sciences, University of Aberdeen, Aberdeen, Scotland, United Kingdom; Aberdeen Cardiovascular & Diabetes Centre, School of Medicine, Medical Sciences & Nutrition, University of Aberdeen, Aberdeen, Scotland, United Kingdom; Ageing Clinical and Experimental Research Team, Institute of Applied Health Sciences, University of Aberdeen, Aberdeen, Scotland, United Kingdom; Aberdeen Cardiovascular & Diabetes Centre, School of Medicine, Medical Sciences & Nutrition, University of Aberdeen, Aberdeen, Scotland, United Kingdom; Ageing Clinical and Experimental Research Team, Institute of Applied Health Sciences, University of Aberdeen, Aberdeen, Scotland, United Kingdom; Department of Biostatistics and Health Informatics, Institute of Psychiatry, Psychology and Neuroscience, King's College London, London, United Kingdom; Aberdeen Cardiovascular & Diabetes Centre, School of Medicine, Medical Sciences & Nutrition, University of Aberdeen, Aberdeen, Scotland, United Kingdom; Ageing Clinical and Experimental Research Team, Institute of Applied Health Sciences, University of Aberdeen, Aberdeen, Scotland, United Kingdom; Aberdeen Royal Infirmary, NHS Grampian, Aberdeen, Scotland, United Kingdom; Ageing Clinical and Experimental Research Team, Institute of Applied Health Sciences, University of Aberdeen, Aberdeen, Scotland, United Kingdom; Department of Nutrition & Dietetics, Birzeit University, Birzeit, West Bank, Palestine; Norwich Medical School, University of East Anglia, Norwich, United Kingdom; Keele Cardiovascular Research Group, Keele University, Stoke-on-Trent, United Kingdom; Aberdeen Cardiovascular & Diabetes Centre, School of Medicine, Medical Sciences & Nutrition, University of Aberdeen, Aberdeen, Scotland, United Kingdom; Ageing Clinical and Experimental Research Team, Institute of Applied Health Sciences, University of Aberdeen, Aberdeen, Scotland, United Kingdom; Aberdeen Royal Infirmary, NHS Grampian, Aberdeen, Scotland, United Kingdom

**Keywords:** malnutrition, stroke and prognosis

## Abstract

**Context and Objective:**

The impact of existing malnutrition on stroke outcomes is poorly recognised and treated. Evidence was systematically reviewed and quantified by meta-analysis.

**Methods:**

MEDLINE, EMBASE and Web of Science were searched from inception to 11 January 2021 and updated in July. Prospective cohort studies, in English, evaluating anthropometric and biomarkers of nutrition on stroke outcomes were included. Risk of bias was assessed using the Scottish Intercollegiate Guidelines Network checklist.

**Results:**

Twenty-six studies (n = 156 249) were eligible (follow-up: One month-14 years). Underweight patients had increased risk of long-term mortality (adjusted hazard ratio = 1.65,1.41-1.95), whilst overweight (0.80,0.74-0.86) and obese patients (0.80,0.75-0.85) had decreased risk compared to normal weight. Odds of mortality decreased in those with high serum albumin (odds ratio = 0.29,0.18-0.48) and increased with low serum albumin (odds ratio = 3.46,1.78-6.74) compared to normal serum albumin (30-35 g/L). Being malnourished compared to well-nourished, as assessed by the Subjective Global Assessment (SGA) or by a combination of anthropometric and biochemical markers increased all-cause mortality (odds ratio = 2.38,1.85-3.06) and poor functional status (adjusted odds ratio = 2.21,1.40-3.49).

**Conclusion:**

Nutritional status at the time of stroke predicts adverse stroke outcomes.

## INTRODUCTION

Cardiovascular disease (CVD) is the leading cause of death worldwide.^1^ Recent improvement in CVD deaths has been slowed by worrying global trends of poor nutrition, increasing obesity and physical inactivity.^2^ Malnutrition is described as a deficiency, excess or imbalance of a wide range of nutrients resulting in a measurable adverse effect on body composition, function and clinical outcome^3^ and can thus be either undernutrition or overnutrition.

Anthropometric tools used to assess nutritional status include body mass index (BMI), triceps skin-fold thickness (TSF), waist circumference (WC), waist-to-hip ratio (WHR) and mid-upper arm circumference (MUAC). BMI is a fast and convenient method of assessing undernutrition (<18.5 kg/m^2^) and overnutrition (overweight 25-29.9 kg/m^2^; obese ≥30kg/m^2^). MUAC evaluates the extent of muscle mass loss due to energy deficiency; patients with moderate/severe undernutrition determined by MUAC compared to normal MUAC have increased mortality risk.^4^ High (>54 g/L) and low (<34 g/L) serum albumin are indicators of protein malnutrition. Diseases involving inflammatory states cause low creatinine levels, which is indicative of malnutrition due to diminished muscle mass.^5^ Another measure, increased serum osmolality which indicates higher concentration of solutes can also reflect dehydration and inadequate fluid intake.

The Subjective Global Assessment (SGA) is a nutritional assessment tool that uses a combination of anthropometric, biochemical and other measures to determine nutritional status, grading patients into A, B or C by evaluating medical history and physical examination.^6^ Similarly, the Malnutrition Universal Screening Tool (MUST) uses such parameters but is instead a nutritional screening tool, identifying individuals at risk of malnutrition by using three components: BMI, weight loss in the last six months and acute disease effect.^7^ Despite the possibility of overestimating high risk and underestimating of moderate risk occurring, this tool has high sensitivity and specificity in addition to a positive predictive value of 0.87 and negative predictive value of 1.0.^8^

The presence of malnutrition in the hospital setting is high; it has been reported that ∼30% of patients are malnourished, whilst this figure varies depending on the patient population and diagnostic criteria.^9^ Malnutrition is reported to be present in 32% of patients six days after an acute stroke.^10^ Dysphagia occurs in almost half of all patients, leading to a 12-fold increase in consequent malnutrition.^11^ Even in patients without dysphagia who have speech, cognitive or visual deficits as a result of a stroke, communication about food inclinations and hunger can be hindered, promoting malnutrition.

To date, no robust evidence exists clearly identifying the relationship between nutritional status at the time of stroke and adverse clinical outcomes. Thus, the aim of this study was to systematically review and meta-analyse the association between standard anthropometric and biochemical measures of malnutrition and nutritional assessment tools in patients with stroke and future outcomes of all-cause mortality, stroke recurrence and poor functional status.

## METHODOLOGY

**Figure 1:**
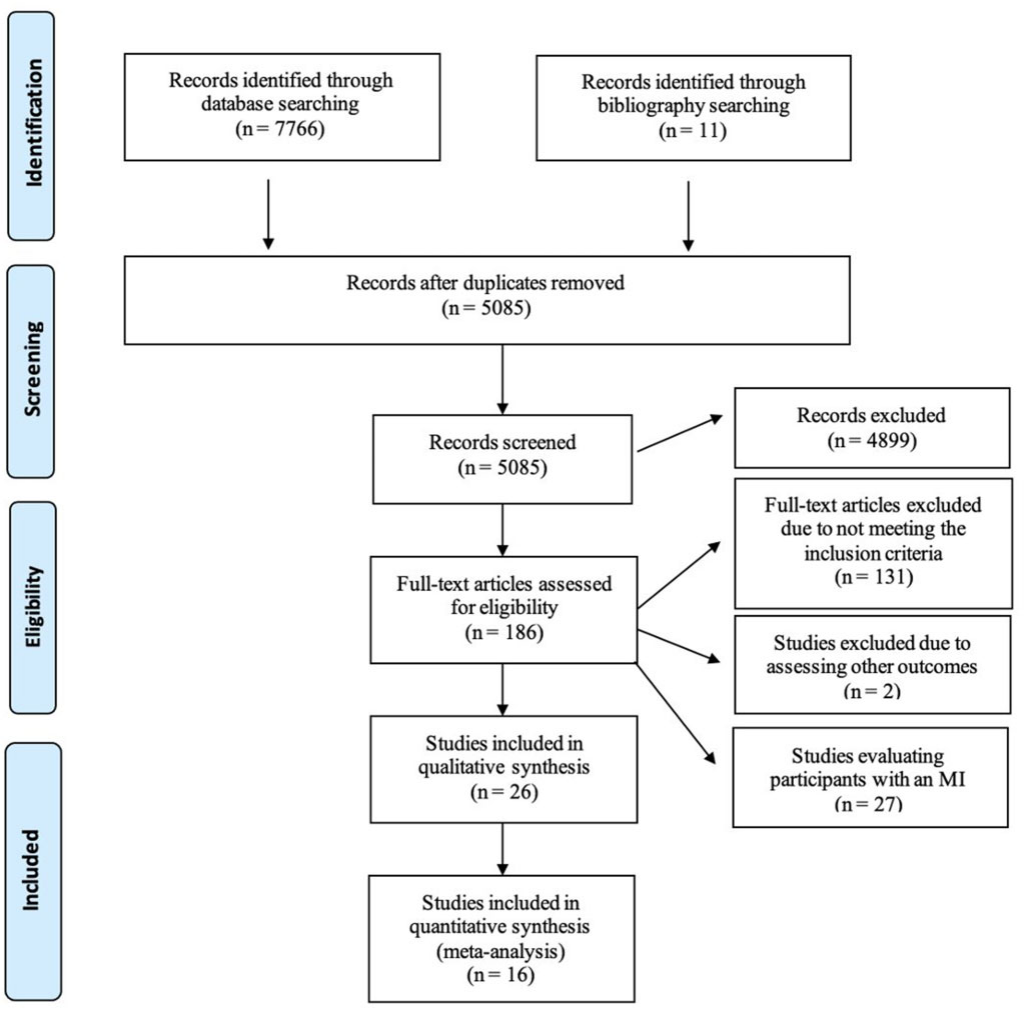
**PRISMA**  **diagram.**

The authors declare that all supporting data are available within the article [and its online supporting information]. This systematic review and meta-analysis was registered in PROSPERO (19 January 2021, registration number: CRD42021231905), in compliance with the Preferred Reporting for Systematic Reviews and Meta-Analyses (PRISMA) guidelines. The full PRISMA data can be found in Figure 1. Although part of a wider search focussing on patients with stroke, MI or TIA, this report focuses solely on stroke outcomes. No other differences between the information registered in PROSPERO and this review exist apart from the inclusion of MI and TIA for the wider search.

### Search strategy

Two reviewers (A.M, L.D.P) conducted independent literature searches using agreed terms on Medline (Ovid), EMBASE (Ovid) and Web of Science. The detailed search strategy for MEDLINE is reported in Figure S1 in the Supporting Information online, where similar but modified terms were used for the other databases. The databases were searched from inception to 11 January 2021 (re-run 13 July 2021). Rayyan review software (https://rayyan.qcri.org/) was used. Bibliographies of eligible papers were examined for further studies.

### Eligibility criteria

Inclusion criteria were: (1) prospective cohort studies, (2) carried out in patients aged 18 years or over who had a myocardial infarction, stroke or transient ischaemic attack, (3) must assess the effect of at least one of serum albumin, serum osmolality, serum creatinine, BMI, weight loss, TSF, WHR, or WC at the time of the event, (4) outcomes including at least one of all-cause mortality, recurrence of cardiovascular event, readmission or poor functional status. Exclusion criteria included studies in asymptomatic coronary heart disease patients, those which included patients younger than 18 years and not written in English. The PICOS (population, intervention, comparator/exposure, outcome, study) table used to construct the research question specifically for stroke can be seen in Table 1.

**Table 1:** PICOS table

	Inclusion	Exclusion
**Patient**	*Adults > 18 years old Stroke*	*Paediatric (< 18 years old)*
**Intervention**	*High or low BMI* *Weight loss* *High or low serum albumin* *High or low serum osmolality* *High or low serum creatinine* *High or low TSF* *High or low WHR* *High or low WC* *Malnourished as judged by SGA or MUST*	*N/A*
**Comparator/Exposure**	*Normal BMI* *No weight loss* *Normal serum albumin* *Normal serum osmolality* *Normal serum creatinine* *Normal TSF* *Normal WHR* *Normal WC* *Well-nourished as judged by SGA or MUST*	*N/A*
**Outcome**	*All-cause mortality* *Stroke recurrence* *Poor functional status*	*N/A*
**Study**	*Prospective Cohort Studies* *Human* *English Language*	*Other study types* *Non-English language*

### Data extraction

Data were extracted onto a standardised data extraction form independently by two reviewers (A.M, L.D.P.). Risk estimates were calculated using ImageJ software from graphical data in the original papers. Data extracted included: Study characteristics (country, year of publication, date of cohort enrolment, follow-up period), subject characteristics (mean age, study population), inclusion and exclusion criteria, type of CVD event, definition of malnutrition, nutrition marker examined, details of intervention and control conditions, outcomes and effect sizes. The extracted data were independently re-assessed by two reviewers before confirming eligibility through discussion and consensus. If disagreement arose, this was resolved by a third reviewer (T.A.P). Any papers with missing information were dealt with by contacting the lead author.

### Quality assessment

Each included paper was critically appraised by two reviewers independently (A.M, L.D.P) using the Scottish Intercollegiate Guidelines Network (SIGN) checklist for Cohort studies. Any disagreement were arbitrated by a third reviewer (T.A.P). Risk of bias was assessed over two sections: Internal validity and overall assessment of the study. Domains in the “internal validity” section included: Subject selection, assessment, study confounders and statistical analysis. The main confounders of age and gender were established; if studies did not account for both, “no” was recorded for the study confounders domain. Studies that met the majority of criteria in the checklist were defined as high (++) quality, whilst studies that met most or only some of the criteria were defined as moderate (+) or low (0) quality, respectively.

### Exposures and outcomes

Patients over the age of 18 diagnosed with a stroke were included, where either one of serum albumin, serum osmolality, serum creatinine, BMI, weight loss, TSF, WHR or WC were measured at admission and compared to the corresponding normal status/exposure. One of the following longitudinal outcomes were also assessed: All-cause mortality, stroke recurrence or poor functional status. The approach of allowing primary studies to define high and low serum albumin concentrations was adopted.

### Data analysis

The association between each nutritional status marker assessed and adverse outcomes of interest was evaluated using meta-analysis whenever possible. For each eligible study, risk estimates and 95% confidence intervals assessing the relationship between nutritional status marker and reported outcome were extracted. Hazard ratios (HR), odds ratios (OR) and mean differences (MD) were not pooled together in the present meta-analysis as statistically, OR, HR and MD are heterogenous summary measures that are used for different types of studies and thus were calculated and presented separately as appropriate. Adjusted risk estimates (by adjusted hazard ratios (aHRs) and adjusted odds ratios (aORs)) were included if a minimum of age and sex were accounted for. Where OR were not presented, unadjusted OR were calculated using available raw data.

Risk estimate specific meta-analyses were performed when possible using an inverse-variance random-effect model. This model assumes that the original studies estimate different, yet related intervention effects, and therefore weights studies based on the mean of a distribution of effect sizes, not allowing one individual study to overly influence the meta-analysis.^12^ In the primary analysis, extreme values of each individual nutrition marker were compared to their corresponding normal value on the outcome of all-cause mortality at any time point after stroke. Secondary analysis examined the same risk for outcomes of readmission, poor functional status and recurrent CV events. The z test was performed for each pooled estimate in the meta-analysis and *P* ≤ 0.05 was considered statistically significant. Different subgroups of the same study were included for each analysis if original studies did not provide an overall risk estimate for the whole group.

### Subgroup analysis to explain heterogeneity

Study heterogeneity was calculated using the inconsistency-value (*I*^2^). We defined *I*^2^ values of 0%, 25%, 50% and 75% as no, low, moderate and high heterogeneity, respectively. If results were heterogeneous, potential sources of heterogeneity were explored by repeating analyses after removing one study at a time. Furthermore, a set of covariates (mean age, follow-up period, study design) were introduced in the model to reduce heterogeneity.

Publication bias was evaluated graphically when possible using funnel plot inspection if there were a sufficient number of studies. Secondary sub-group analyses by age, sex and CVD event severity were planned but was not undertaken due to insufficient data. All meta-analyses were undertaken using RevMan Version 5.4.^13^

## RESULTS

Of 7,766 records from initial search, 5085 were retrieved after removal of duplicates. Of them, 186 studies were deemed eligible for full-text review, with 53 matching the eligibility criteria. From 53 studies, 27 involved a population of participants with an MI and were excluded from this report. Finally, 26 studies (n = 156 249) were included in this systematic review which focuses on stroke outcomes, of which 16 (n = 62 243) were eligible for meta-analysis (Figure 1).

### Study characteristics

The descriptive characteristics of included studies are displayed in Table 2.^14–34^

**Table 2:** Study Characteristics of all included studies in this review

Study	Follow up (months)	Females/Males	Country	Exposure	Comparison	Outcome Assessed
*Anthropometric Nutrition Markers*						
Andersen & Olsen 2013^14^	117.6	18098/20408	Denmark	BMI ≥30, 25.0–29.9, <18.5 kg/m^2^	BMI 18.5–24.9 kg/m^2^	Recurrent event
Andersen & Olsen 2015^15^	117.6	14076/15250	Denmark	BMI ≥30, 25.0–29.9, <18.5 kg/m^2^	BMI 18.5–24.9 kg/m^2^	Mortality, readmission
Bell 2013^16^	120	3173 females^a^	USA	BMI ≥30, 25.0–29.9, <18.5 kg/m^2^	BMI 18.5-24.9 kg/m^2^	Mortality
Branscheidt 2016^17^	3	361/535	Switzerland	BMI ≥35, 30.0-34, 25.0-29.9, <18.5 kg/m^2^	BMI 18.5-24.9 kg/m^2^	Mortality, functional status
Jang 2015^18^	6	770/1287	South Korea	BMI ≥30, 25-30, 23-25, <18.5 kg/m^2^	BMI 18.5-23 kg/m^2^	Functional status
Kawase 2016^19^	31.2	446/760	Japan	BMI ≥ 25, <18.5 kg/m^2^	BMI 18.5–24.9 kg/m^2^	Mortality, recurrent event
Kim 2012^20^	90	14540/19592	South Korea	BMI≥32.5, 30.0-32.4, 27.5-29.9, 25.0-27.4, 23.0-24.9, 18.5-19.9, <18.5 kg/m^2^	BMI 20.0-22.9 kg/m^2^	Mortality
Leszczak 2019^21^	3	42/58	Poland	BMI ≥30, 25–29.99, ≤18.49 kg/m^2^	BMI 18.5–24.99 kg/m^2^	Functional status
Olsen 2008^22^	60	10504/11380	Denmark	BMI ≥35, 30.0-34, 25.0-29.9, <18.5 kg/m^2^	BMI 18.5–24.9 kg/m^2^	Mortality
Ryu 2011^23^	48	541/1051	Korea	BMI >30, 25-29.9, 23-25, <18.5 kg/m^2^	BMI 18.5–23 kg/m^2^	Mortality
Skolarus 2013^24^	170	913/878	USA	BMI ≥40, 35–39.9, 30–34.9, <18.5 kg/m	BMI 25–29.9 kg/m^2^	Mortality
Vemmos 2011^25^	120	1050/1735	Sweden	BMI ≥30, 25–29.9 kg/m^2^	BMI <25 kg/m^2^	Mortality
Wang 2020^26^	75	295/459	China	BMI > 28, 24–28, <18.5 kg/m^2^	BMI 18.5 - <24 kg/m^2^	Mortality
Zhao 2013^27^	3	4136/6769	China	BMI >32.5, 27.5-32.4,23-27.4, <18.5 kg/m^2^	BMI 18.5–22.9 kg/m^2^	Mortality
*Biochemical Nutrition Markers*						
Abubakar 2013^28^	1	36/39	Nigeria	<35, >35 g/L	30-35 g/L	Mortality
Bhalla 2000^29^	3	87/80	UK	>296 mOsm/kg	<296 mOsm/kg	Mortality, functional status
Carter 2007^30^	88.8	271/274	UK	>38 g/L; >117, 98-117, 82-97 mmol/l	<38 g/L; <82 mmol/l	Mortality
Gariballa 1998a^31^	3	129/96	UK	≥35 g/L	<35 g/L	Mortality
Gariballa 1998b^32^	3	180/81	UK	<35 g/L	≥35 g/L	Mortality
Idicula 2009^33^	24	195/249	Norway	<40, >45 g/L	40-45 g/L	Mortality
Pandian 2011^34^	6	167/281	India	<35 g/L	35-50 g/L	Functional status
Zhang 2016^35^	12	294/398	China	>40.8, 38.8-40.8, 36.7-38.8, ≤36.7 g/L	≤36.7 g/L	Mortality, recurrent event
*Nutrition Assessment Tools*						
Davalos 1996^36^	3	37/67	Spain	Undernutrition^f^	Well-nourished^d^	Functional status
Davis 2004^37^	1	87/98	Australia	Undernutrition^c^	Well-nourished^d^	Functional status, mortality
FOOD Trial 2003^38^	6	1492/1520	Australia, Belgium, Brazil, Canada, Czech Republic, Denmark, Hong Kong, India, Italy, New Zealand, Poland, Portugal, Republic of Ireland, Turkey, UK	Undernutrition^e^	Well-nourished^e^	Functional status, mortality
Gomes 2016^39^	6	263/274	UK	High, medium risk^b^	Low risk^b^	Mortality
Pandian 2011^34^	1	167/281	India	Undernutrition^c^	Well-nourished^d^	Functional status

a Study included females only.

b The sum of scores obtained for each question related to BMI, unintentional weight loss, the effect of acute disease, and the inability to eat for more than five days results in an overall risk of malnutrition score (MUST), which categorises patients into low (score = 0), medium (score = 1) or high risk (score > 2).

c Malnourished defined by SGA: Rating B or C by the SGA for nutritional status.

d Well-nourished defined by SGA: Rating A by the SGA for nutritional status.

e Malnourished and well-nourished defined by clinician judgement on the basis of either their own beside assessment or, when practical, from a fuller assessment including weight, height, dietary history and blood tests.

f Malnourished defined by if serum albumin was <35 g/L or if TSF or MAMC was less than the 10th centile of the reference population (TSF <59.5% and 62.5% and MAMC (midarm muscle circumference) <85% and 86.4% for men and women, respectively).

#### Anthropometric markers.

Of the 14 prospective cohort studies examining the relationship between BMI and outcomes, 149 107 participants (46% females) diagnosed with stroke were included.^14–27^ Mean age across the studies ranged from 54.2 to 78.0 years. All-cause mortality was determined at three months for three studies^17^^,^^21^^,^^27^ at six months for one study^18^ and at 12 months or longer in the remaining ten studies.^14–16^^,^^19^^,^^20^^,^^22–26^ Poor functional status was ascertained by the modified Rankin Scale (mRS), Barthel Index (BI) and Functional Independence Measure. This was measured at three months in two studies^16^^,^^20^ and at six months in one study.^18^ Readmission and stroke recurrence was measured in one^14^ and two studies, respectively.^14^^,^^19^

#### Biochemical markers.

Eight studies investigated the association between biochemical markers and adverse outcomes.^28–35^ 2857 participants (48% female) with stroke were included. Serum albumin was measured in six,^28^^,^^30–35^ serum creatinine in one^30^ and serum osmolality^31^ in another, with all exposures being measured by blood samples and biochemical analysis at different time-points. Regarding adverse outcomes, all-cause mortality was determined in one study at one month,^28^ three at three months,^29^^,^^31^^,^^32^ one at six months^34^ and in three at 12 months or longer.^30^^,^^34^^,^^35^ Poor functional status was measured in one study at three months by BI^29^ and in the other at six months by mRS.^34^ Stroke recurrence was measured in one study.^35^

#### Nutritional assessment tools.

Five studies reported the relationship between nutritional assessment tools and outcomes in 4286 participants (48% female).^34–39^ Two studies used SGA^34^^,^^37^, a validated nutritional assessment tool while one study used MUST^39^, a nutritional screening tool. Two studies used a combination of anthropometric and biochemical markers, one of which used clinical judgment or blood tests^38^ and the other a combination of MUAC, TSF and albumin.^36^ Regarding the measured outcome, three studies assessed all-cause mortality, one at one month^37^ and two at six months.^39^ Poor functional status was measured by the mRS in three studies^36–38^ and by BI in one study.^34^

### Assessment of risk of bias in included studies

The results of critical appraisal are presented in Table S1 in the Supporting Information online. Overall, the studies were of moderate to high methodological quality except one.^19^ Common strengths were: Clear and focused question (n = 26; 100%),^14–39^ selecting of participants (n = 26; 100%)^14–39^ clearly defining the number of participants in each group (n = 24; 92%),^14–30^^,^^33–39^ distinctly stating outcomes (n = 26; 100%)^14–39^ and reliable exposure assessment (n = 25; 96%).^14–37^^,^^39^ The majority of studies had acceptable drop-out rates (n = 22; 85%),^14–18^^,^^20^^,^^22–27^^,^^29–37^^,^^39^ provided confidence intervals (n = 23; 88%)^14–18^^,^^20–23^^,^^25–35^^,^^37–39^ or found a clear association between exposure and outcome (n = 23; 88%).^14–16^^,^^18–37^ The follow-up was suitably extensive (ie, six months or longer) in 67% of studies that assessed mortality^14–16^^,^^18–26^^,^^28–36^^,^^38^^,^^39^ and in all of studies that assessed recurrent events and readmission.^14^^,^^15^^,^^35^ In studies that assessed poor functional status following a stroke, follow-up was suitably extensive (ie, 3 mo or longer) in 78%.^17^^,^^18^^,^^21^^,^^29^^,^^34^^,^^36^^,^^38^ Four studies (15%)^18–39^ adjusted for only one major confounding variable (either age or sex) and five studies (19%)^14–34^ did not adjust for either. Twenty-two studies (85%) did not blind assessors to the exposure status.^14–24^^,^^26^^,^^28–32^^,^^34–37^^,^^39^

### Primary outcome: Association between nutritional status and all-cause mortality

#### BMI

**Figure 2:**
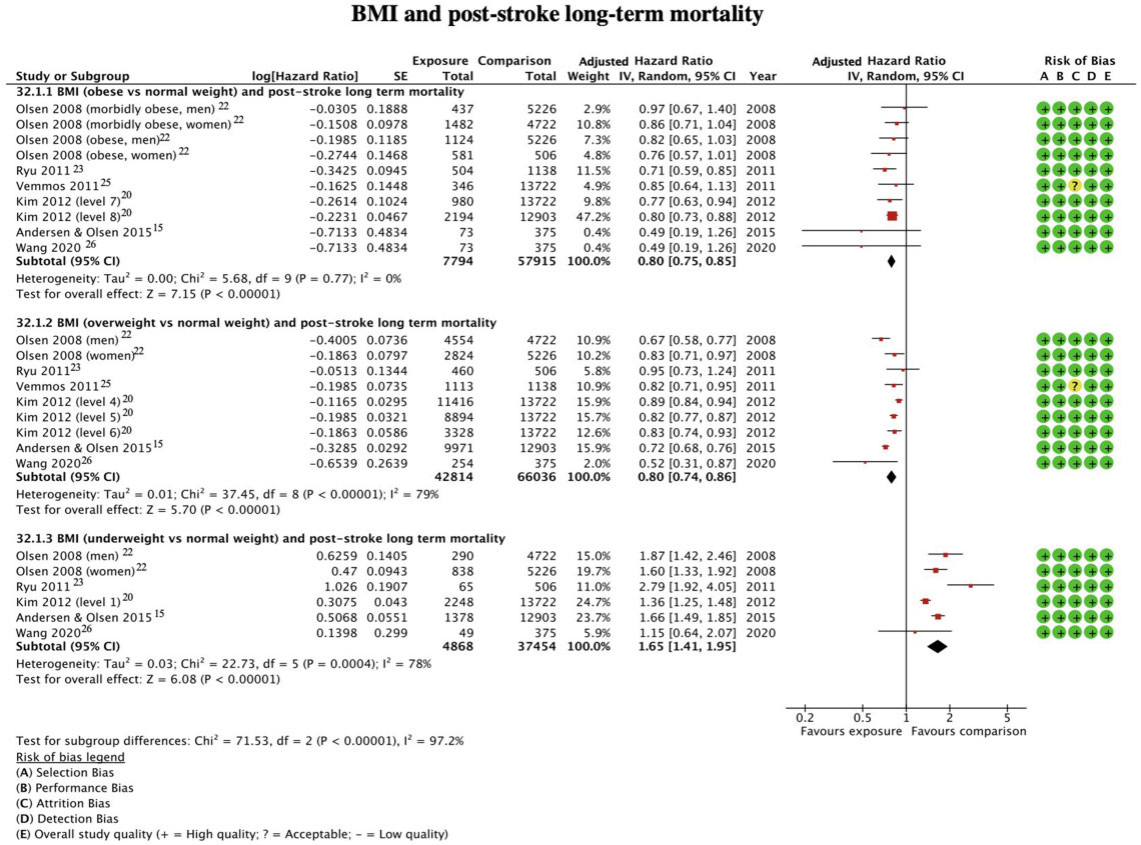
**BMI and post-stroke long-term mortality**.

Six studies (n = 6052) assessed outcomes in obese patients (Figure 2).^15^^,^^20^^,^^22^^,^^23^^,^^25^^,^^26^ Two studies^15^^,^^26^ presented both unadjusted and adjusted hazard ratios and four studies^20^^,^^22^^,^^23^^,^^25^ presented only adjusted hazard ratios. Obesity was a significant predictor of all-cause mortality, with the risk of death being 20% lower in obese compared to normal weight patients aHR = 0.80 (95%CI 0.75-0.85, *P* < 0.0001, *I*^2^ = 0%).

Overweight patients were evaluated in six studies, assessing all-cause mortality in 49,578 participants (Figure 2).^15^^,^^20^^,^^22^^,^^23^^,^^25^^,^^26^ Two studies^15^^,^^26^ presented both unadjusted and adjusted hazard ratios and four studies^20^^,^^22^^,^^23^^,^^25^ presented only adjusted hazard ratios. Being overweight was a significant predictor of death with the risk of all-cause mortality being 20% lower in overweight compared to normal weight patients aHR = 0.80 (95%CI 0.74-0.86, *P* < 0.0001, I^2^ = 79%).

It was possible to assess the risk of mortality in underweight patients in five studies (n = 1948).^15^^,^^20^^,^^22–24^^,^^26^ Two studies^13^^,^^26^ presented both unadjusted and adjusted hazard ratios and three studies^20^^,^^22^^,^^23^ presented only adjusted hazard ratios. The results indicated that being underweight was a significant predictor of all-cause mortality (Figure 2), with the risk of death being 65% higher in underweight compared to normal weight patients aHR = 1.65 (95%CI 1.41–1.95, *P* = 0.0004, I^2^ = 78%).

#### Serum albumin

**Figure 3:**
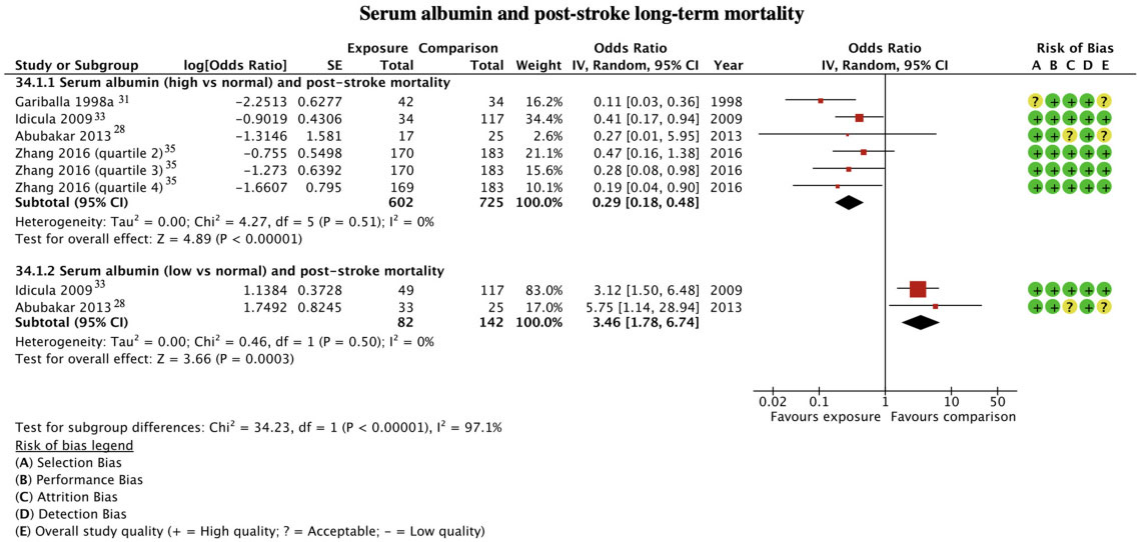
**Serum albumin and post-stroke long-term mortality**.

A comparison of mortality risk was possible in patients with high serum albumin from four studies (Figure 3).^27^^,^^31^^,^^33^^,^^35^ From a population of 602 participants, patients with high serum albumin levels at the time of a stroke demonstrated a 71% decreased odds of all-cause mortality compared with normal serum albumin levels OR = 0.29 (95%CI 0.18–0.48, *P* < 0.0001, I^2^ = 0%).

Data from two studies (n = 142) were used in the analysis of patients with low serum albumin compared to normal serum albumin at the time of a stroke (Figure 3).^28^^,^^33^ Patients with low serum albumin had 246% increased odds of all-cause mortality OR = 3.46 (95%CI 1.78–6.74, *P* = 0.0003, I^2^ = 0%).

#### Nutritional assessment tools

**Figure 4:**
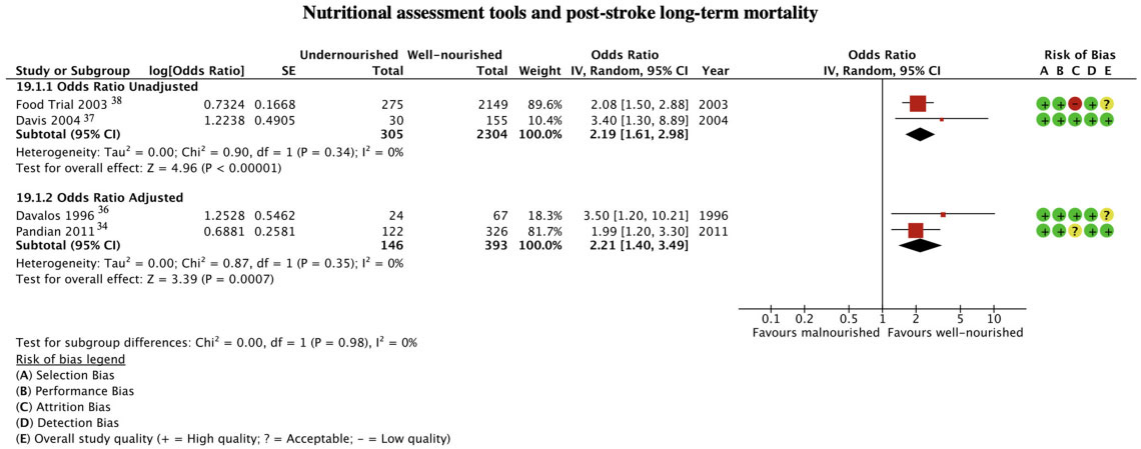
**Nutritional assessment tools and post-stroke long-term mortality**.

The impact on mortality of being malnourished at the time of a stroke, as judged by the SGA (grade B or C) or by a combination of anthropometric and biochemical markers was evaluated in four studies with 451 participants (Figure 4).^34^^,^^36–38^ One study evaluated a nutritional screening tool (MUST) and used a different risk estimate, hence not being eligible for meta-analysis.^39^ People who were malnourished had 121% increased odds of death compared with those who were well-nourished on admission following a stroke aOR = 2.21 (95%CI 1.40–3.49, *P* = 0.0007, I^2^ = 0%).

### Secondary outcomes

Two studies^14^^,^^19^ evaluated the impact of a low or high BMI on stroke recurrence, whilst one study^19^ investigated the risk of stroke recurrence in individuals with high serum albumin, making this insufficient for meta-analysis. Similarly, only one study was identified for the secondary outcome of readmission to hospital.^35^

### Association between nutritional status and poor functional status

#### BMI

Two studies with 795 participants provided data for analysis on poor functional status in obese patients (see Figure S2 in the Supporting Information online).^18^^,^^21^ There was no statistically significant difference in the risk of poor functional status in those classified as obese compared with normal weight individuals MD = 2.86 (95%CI -0.28-6.00, *P* = 0.07, I^2^ = 53%).

Two studies presented data on overweight patients at the time of a stroke and poor functional status (n = 627; see Figure S3 in the Supporting Information online).^18^^,^^21^ Meta-analysis revealed no significant relationship in the risk of poor functional status for overweight compared to normal weight patients MD = 0.50 (95%CI -2.69-3.69, *P* = 0.76, I^2^ = 35%).

#### Nutritional assessment tools

Being malnourished, as judged by the SGA (grade B or C) or by a combination of anthropometric and biochemical markers was a statistically significant indicator of poor functional status following stroke in four studies (n = 451).^24^^,^^26–28^ Unadjusted and adjusted odds ratios were present for two studies each (see Figure S4 in the Supporting Information online). The pooled results indicated that being malnourished compared to well-nourished resulted in 121% greater odds of poor functional status aOR = 2.21 (95%CI 1.40–3.49, *P* = 0.0007, I^2^ = 0%).

### Heterogeneity, sensitivity analyses and publication bias

Meta-analysis of studies investigating all-cause mortality and malnourished (judged by SGA or by a combination of anthropometric and biochemical markers), obese and high serum albumin patients showed no significant heterogeneity. Results from studies involving participants who were overweight or underweight had high heterogeneity. Sensitivity analyses showed reduced heterogeneity from high to moderate, with a minor change in effect size after removal of one study with a different study design (see Figure S5 in the Supporting Information online).^23^

In studies of poor functional status, meta-analysis of studies involving malnourished participants (judged by SGA or by a combination of anthropometric and biochemical markers) revealed no significant heterogeneity, while those of obese and overweight participants showed moderate and low heterogeneity, respectively. No sensitivity analyses were therefore conducted for the outcome of poor functional status.

No publication bias was evident, as observed by the funnel plot for BMI and long-term mortality available in Figure S6 in the Supporting Information online.

## DISCUSSION

To the best of this author’s knowledge, this is the first systematic review and meta-analysis investigating the relationship between nutritional status, as judged by anthropometric and biochemical markers and nutritional assessment tools, at the time of a stroke and adverse outcomes. The vast majority (25 out of 26 included studies) were of moderate to high quality. Results showed a decreased risk of mortality in obese, overweight and high serum albumin patients compared to normal weight and normal serum albumin, respectively, and an increased risk in underweight, malnourished (judged by SGA or by a combination of anthropometric and biochemical markers) and low serum albumin patients compared to normal weight, well-nourished and normal serum albumin, respectively. Similarly, patients who were malnourished (judged by SGA or by a combination of anthropometric and biochemical markers) had an increased risk of poor functional status compared to well-nourished patients.

The results of this study are consistent with findings of studies exploring the “obesity paradox.”^40^ Activation of catabolic pathways post-stroke occurs from a combination of causes, such as impaired feeding, infection and stroke-related sarcopenia.^41^ An increased metabolic reserve present in patients with excess body fat may hence cause a diminished effect from this adverse dysregulation. Other mechanisms behind the paradox are proposed to be the storage of toxic lipophilic chemicals in adipose tissue and weight loss caused by a deterioration in condition that drives the BMI of obese patients into the normal weight category.^42^

These data, however, need to be interpreted with caution. First, although age and sex were accounted for in most of the included studies, BMI may have lost its significance as a predictor of all-cause mortality if corrected for several co-morbidities present in the obese population.^43^ Furthermore, underweight or normal weight patients in this review were mostly older patients, hence increasing mortality risk by virtue of age; whilst age was adjusted, there may be a residual confounding effect. Evidence also suggests that patients with higher BMI were younger and had a smaller infarct size, thus perhaps less severe stroke.^44^ Only seven individual studies in the present meta-analysis adjusted for stroke severity, thus potentially distorting our study results.^22^^,^^24^^,^^25^^,^^27–29^^,^^34^ Additionally, findings of this review rely on the measurement of obesity by BMI. The diagnostic performance of BMI has been widely disputed, with a decline in accuracy detected with increasing age.^45^ This method also cannot distinguish between body fat percentage and lean mass or measure an individual’s cardio-respiratory fitness (CRF), which is an important mediator of CVD risk than obesity due to the effect of obesity being reduced in individuals with a normal or high CRF.^46^ The use of tools such as waist circumference, a more precise measure of central obesity, has disproven the existence of the obesity paradox.^47^ Despite these limitations, BMI is reasonably easy to measure and from a prognostic point of view, its value in identifying at risk patients with stroke who are likely to be linked to poor outcomes has been confirmed through the present systematic review.

All included studies found that underweight patients had an increased risk of all-cause mortality following stroke. It is hypothesised that frailty mediates the association between undernutrition and adverse outcomes post-stroke. Patients who are underweight are more vulnerable to experiencing frailty, which itself is linked to increases in both the incidence of CVD events^48^ and adverse outcomes of mortality post-stroke^49^ because of reduced physical reserve.

The findings of this review also suggest a two-fold increased risk of all-cause mortality and poor functional status in those who were malnourished (judged by SGA or by a combination of anthropometric and biochemical markers) at the time of a stroke. Similar findings have been reported in retrospective cohort studies when using the SGA.^50^ However, of note, two included studies which used the combination of anthropometric and biochemical markers did not use a validated tool. One study used “clinician judgement on the basis of either their own bedside assessment or, when practical, from a fuller assessment that might include weight, height, dietary history or blood tests”.^38^ The other study determined that an individual was malnourished if either serum albumin was <35 g/L or if the TSF or MAMC was less than the 10^th^ centile of their reference population (TSF <59.5% and 62.5% and MAMC <85% and 86.4% for men and women, respectively).^36^ A combination of variables can aid in determining malnutrition, such as weight change, BMI and muscle wasting. In isolation, these measures have limited use but used together, through validated assessment tools or combinations of anthropometric and biochemical measures, dramatically increases reliability and accuracy.

Serum albumin is already decreased in the acute inflammatory state such as in stroke^51^ and thus, this may not be an accurate reflection of an individual’s nutritional status. BAPEN (British Association for Parenteral and Enteral Nutrition) state that a low level of serum albumin may indicate inflammation or infection is present and therefore should not be used to determine nutritional status.^52^ Though serum albumin may not be of clinical significance, low levels could indicate the need for a complete and detailed nutritional assessment and potentially be a link to poorer outcomes in individuals who have suffered stroke. It is also important to highlight the differences between nutritional assessment and nutritional screening. Nutritional assessment is the process of “collecting and interpreting information in order to make decisions about the nature and cause of nutrition related health issues”, whilst nutritional screening “can be carried out by any healthcare professional and may lead to a nutritional assessment by a dietician”. ^52^ Therefore, the MUST score, a tool for nutritional screening, was not included in the meta-analysis of nutritional assessment tools. Nevertheless, the inclusion of both tools in the overall review allowed the assessment of a wider range of nutritional markers in the identification of patient groups at risk of adverse outcomes after stroke. These results should therefore be interpreted with caution. Other studies^55^^,^^56^ which used other nutritional screening tools such as the MNA-SF^53^ and nutritional assessment tools such as the PG-SGA^54^ did not meet our predefined inclusion criteria. Although this may have been of some interest, available studies are nevertheless cross-sectional^55^ or without numerical estimates.^56^ This perhaps indicates the lack of literature evaluating their relationship with longitudinal outcomes of stroke. Further studies are hence required to strengthen the understanding of this association.

Although evidence was found supporting the “obesity paradox,” it is important to consider the range of limitations of BMI as well as the potential for confounding. These findings should not undermine the importance of proper lifestyle management or promote the wrong public health message regarding obesity. Nutritional assessment tools clearly provide a comprehensive assessment of nutrition and are able to determine the risk or presence of malnutrition, which serves as a prognostic marker of all-cause mortality and poor functional status. Early detection could promote dietary nutritional therapy and be a definitive method of preventing adverse clinical outcomes in malnourished patients. Timely nutritional therapy in stroke patients significantly improves (*P* < 0.001) the efficiency of rehabilitation.^57^ Improvements have been ascribed to higher caloric intake improving basal metabolism, with subsequent rises in protein intake aiding improvement in immune status and neuronal survival. Despite current National Institute for Health and Care Excellence guidelines recommending screening with a validated tool, malnutrition is often undiagnosed and untreated in stroke patients.^58^ This causes a host of problems such as slower immune response, reduced muscle mass, mental health problems and impaired development that can lead to adverse outcomes.^34^ Early identification of patients at high risk, evident by results of this study can therefore promote early treatment and hence support prevention of such factors that lead to increased morbidity and mortality.

This systematic review and meta-analysis has several strengths, one of which was the inclusion of only prospective cohort studies to reduce the reverse causality and establish the temporal trends in exposure and outcome relationship. A comprehensive search strategy and bibliography searching aided the generalisability of findings. Analysis by risk estimate allowed meta-analysis to be conducted for all presented estimates. Although this study is the first of its kind, certain limitations should be noted. First, no grey literature was included. Second, variation in length of follow-up of included studies from one-month to 14 years may have affected true risk estimates. Third, high heterogeneity was present in most analyses, suggesting variation in study design, population or outcome and exposure measures. Fourth, due to the inclusion of only baseline nutritional markers, meta-analysis would not account for the effect of change in exposure on outcomes, raising the possibility of reverse causation. Finally, this review was limited by original studies with regards to known confounders which might not have been adequately adjusted for, indicating that the presence of collider bias may have distorted the association between exposure and outcome due to unmeasured confounders.

## CONCLUSION

To sum up, for the first time, a clear prospective association between nutritional status at the time of stroke and subsequent adverse clinical outcomes is reported. Due to the reliance of utilising BMI to evaluate malnutrition in the existing literature, future research should address methodological limitations of previous studies, such as deficiencies in measuring potentially useful nutritional prognostic markers as well as measuring changes in an exposure and lack of adequate adjustment for comorbidities and stroke severity. Nevertheless, findings suggest that clinicians should utilise nutritional assessment tools to diagnose malnutrition and initiate early nutritional therapy in stroke patients, to potentially prevent adverse outcomes.

## Supplementary Material

nuac034_Supplementary_Data

## Supporting Information

The following Supporting Information is available through the online version of this article at the publisher’s website.

*Figure S1*  **MEDLINE Search Strategy**

*Figure S2*  **Obesity and Post-Stroke Poor Functional Status**

*Figure S3*  **Overweight and Post-Stroke Poor Functional Status**

*Figure S4*  **Malnourished and Post-Stroke Poor Functional Status**

*Figure S5*  **Sensitivity Analysis of Underweight and Post-Stroke Long-Term Mortality**

*Figure S6*  **BMI and Post-Stroke Long-Term Mortality Funnel Plot**

*Table S1*  **Critical Appraisal**

*Table S2*  **Studies not Included for Meta-Analysis**

*Table S3*  **Study Results**

*Table S4*  **PRISMA Checklist**

## ABBREVIATIONS AND ACRONYMS

aHR — Adjusted Hazard Ratio
aOR — Adjusted Odds Ratio
BMI — Body Mass Index
CVD — Cardiovascular Disease
HR — Hazard Ratio
MD — Mean Difference
MUAC — Mid-Upper Arm Circumference
MUST — Malnutrition Universal Screening Tool
OR — Odds Ratio
SGA — Subjective Global Assessment
TSF — Triceps Skinfold Thickness
WC — Waist Circumference
WHR — Waist-to-Hip Ratio
