# Supplementary material for: The relationship between nutritional status at the time of stroke on adverse outcomes: a systematic review and meta-analysis of prospective cohort studies
Source: Nutr Rev. 2022 May 27;80(12):2275–87. doi: 10.1093/nutrit/nuac034 (PMC9647329; doi:10.1093/nutrit/nuac034)
Supplement: nuac034_Supplementary_Data [file nuac034_supplementary_data.docx]

Supporting Information

Figure S1

Figure 1: Search Strategy for MEDLINE


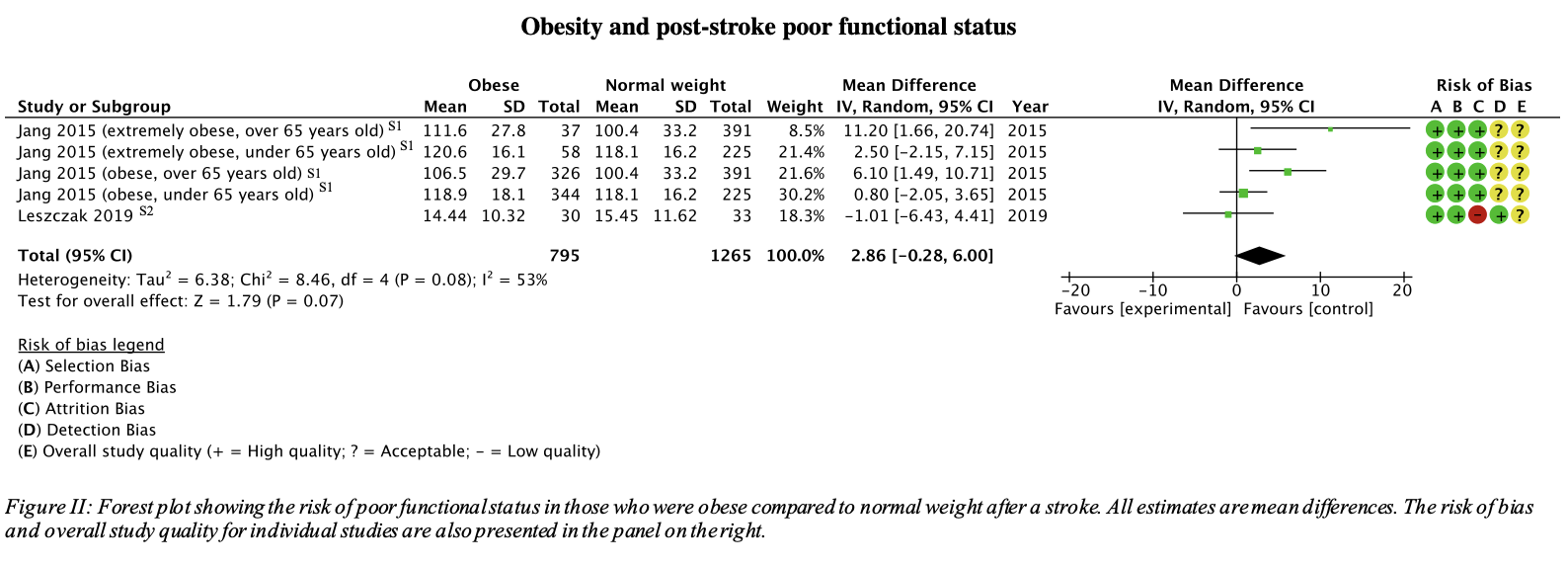
Figure S2

S1 - Jang SY, Shin Y il, Kim DY, et al. Effect of obesity on functional outcomes at 6 months post-stroke among elderly Koreans: A prospective multicentre study. BMJ Open. 2015;5(12). doi:10.1136/bmjopen-2015-008712

S2 - Leszczak J, Czenczek-Lewandowska E, Przysada G, et al. Association between body mass index and results of rehabilitation in patients after stroke: A 3-month observational follow-up study. Medical Science Monitor. 2019;25. doi:10.12659/MSM.915586

Figure S3


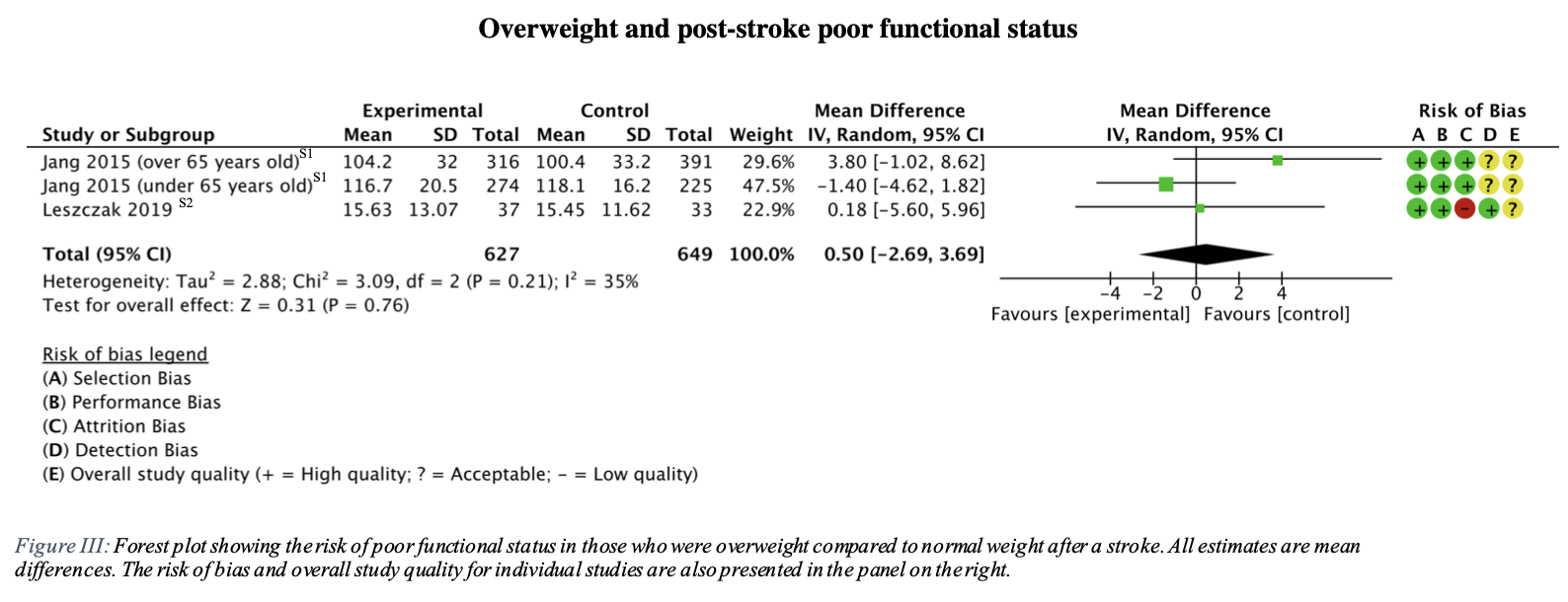


S1 - Jang SY, Shin Y il, Kim DY, et al. Effect of obesity on functional outcomes at 6 months post-stroke among elderly Koreans: A prospective multicentre study. BMJ Open. 2015;5(12). doi:10.1136/bmjopen-2015-008712

S2 - Leszczak J, Czenczek-Lewandowska E, Przysada G, et al. Association between body mass index and results of rehabilitation in patients after stroke: A 3-month observational follow-up study. Medical Science Monitor. 2019;25. doi:10.12659/MSM.915586


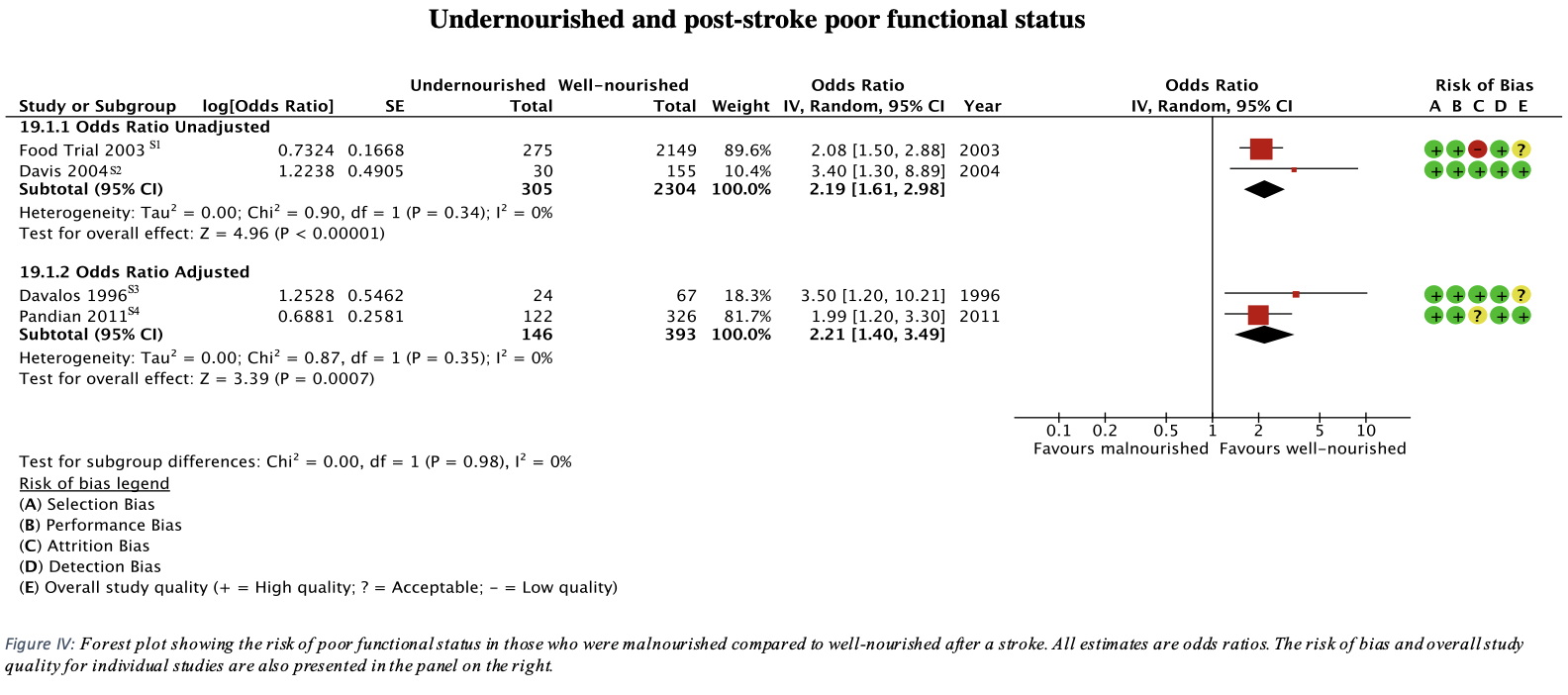
Figure S4

S1 - FOOD Trial. Poor nutritional status on admission predicts poor outcomes after stroke observational data from the food trial. Stroke. 2003;34(6). doi:10.1161/01.STR.0000074037.49197.8C

S2 - Davis JP, Wong AA, Schluter PJ, Henderson RD, O’Sullivan JD, Read SJ. Impact of premorbid undernutrition on outcome in stroke patients. Stroke. 2004;35(8). doi:10.1161/01.STR.0000135227.10451.c9

S3 - Davalos A, Ricart W, Gonzalez-Huix F, et al. Effect of malnutrition after acute stroke on clinical outcome. Stroke. 1996;27(6). doi:10.1161/01.STR.27.6.1028

S4 - Pandian JD, Jyotsna R, Singh R, et al. Premorbid nutrition and short term outcome of stroke: A multicentre study from India. Journal of Neurology, Neurosurgery and Psychiatry. 2011;82(10). doi:10.1136/jnnp.2010.233429

Figure S5


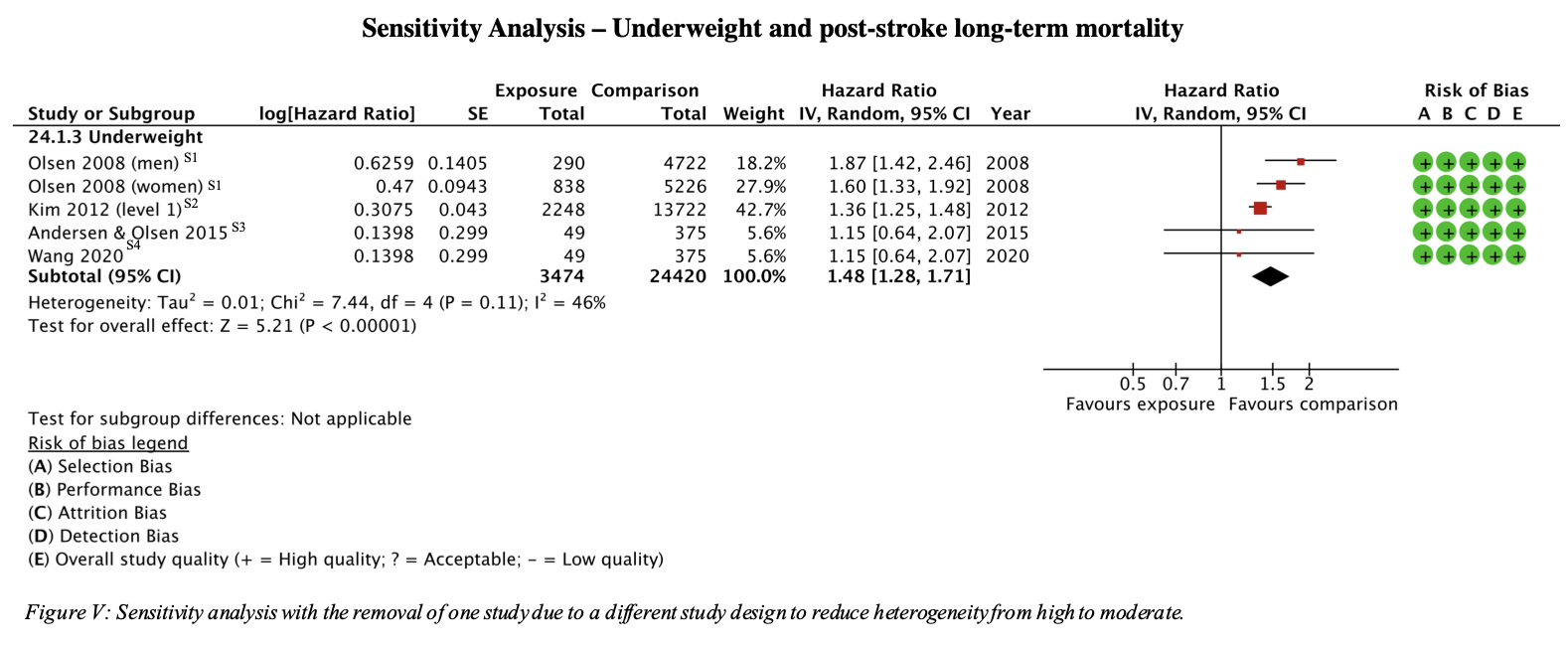


S1 - Olsen TS, Dehlendorff C, Petersen HG, Andersen KK. Body mass index and poststroke mortality. Neuroepidemiology. 2008;30(2). doi:10.1159/000118945

S2 - Kim BJ, Lee SH, Jung KH, Yu KH, Lee BC, Roh JK. Dynamics of obesity paradox after stroke, related to time from onset, age, and causes of death. Neurology. 2012;79(9). doi:10.1212/WNL.0b013e318266fad1

S3 - Andersen KK, Olsen TS. The Obesity Paradox in Stroke: Lower Mortality and Lower Risk of Readmission for Recurrent Stroke in Obese Stroke Patients. International Journal of Stroke. 2015;10(1). doi:10.1111/ijs.12016

S4 - Wang J, Li J, Li M, et al. Association between dynamic obesity and mortality in patients with first-ever ischemic stroke: A hospital-based prospective study. Medicine. 2020;99(38). doi:10.1097/MD.0000000000022243

Figure S6

Figure VI: Funnel plot for BMI and post-stroke long term mortality.

Table S1


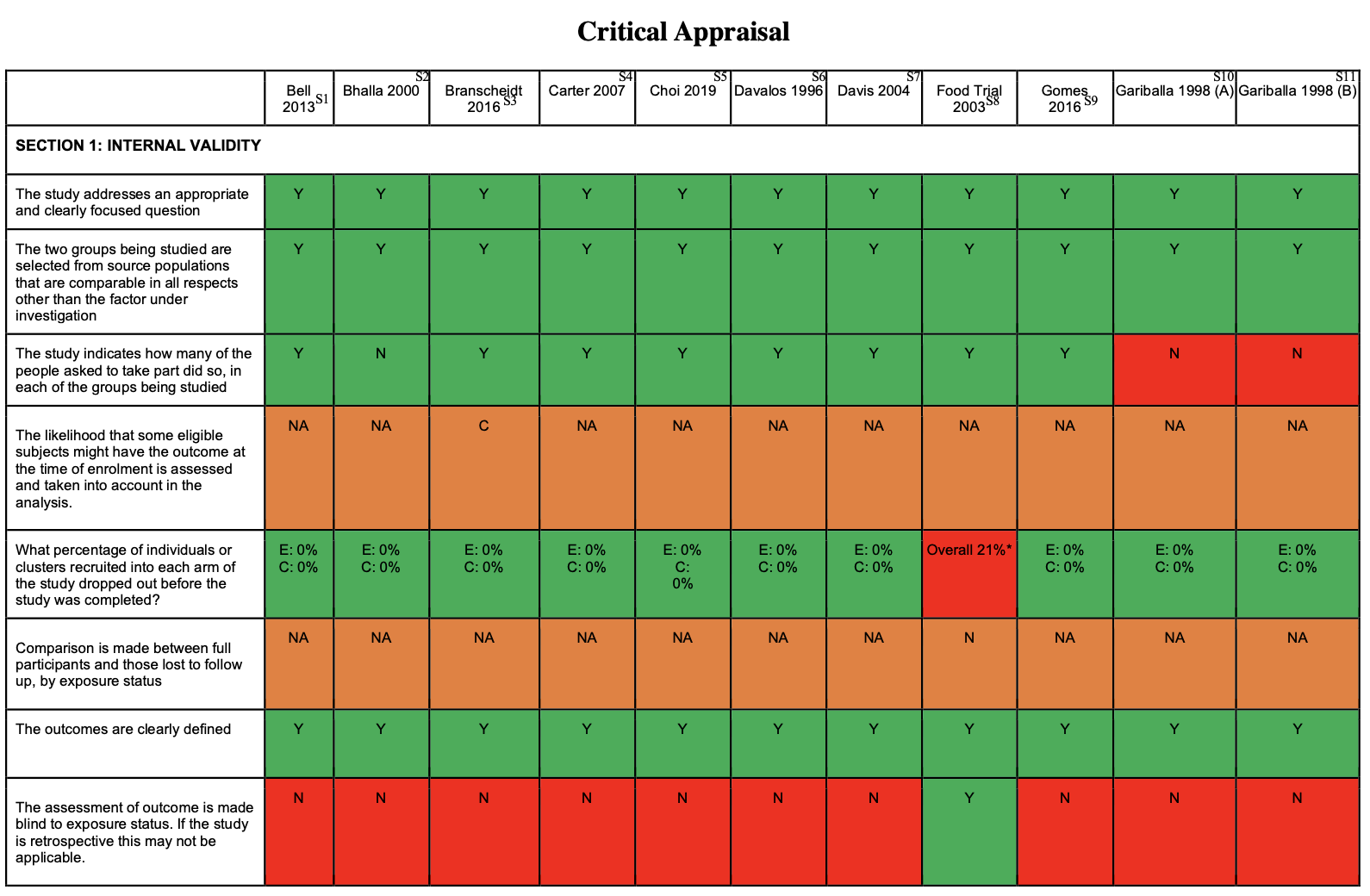

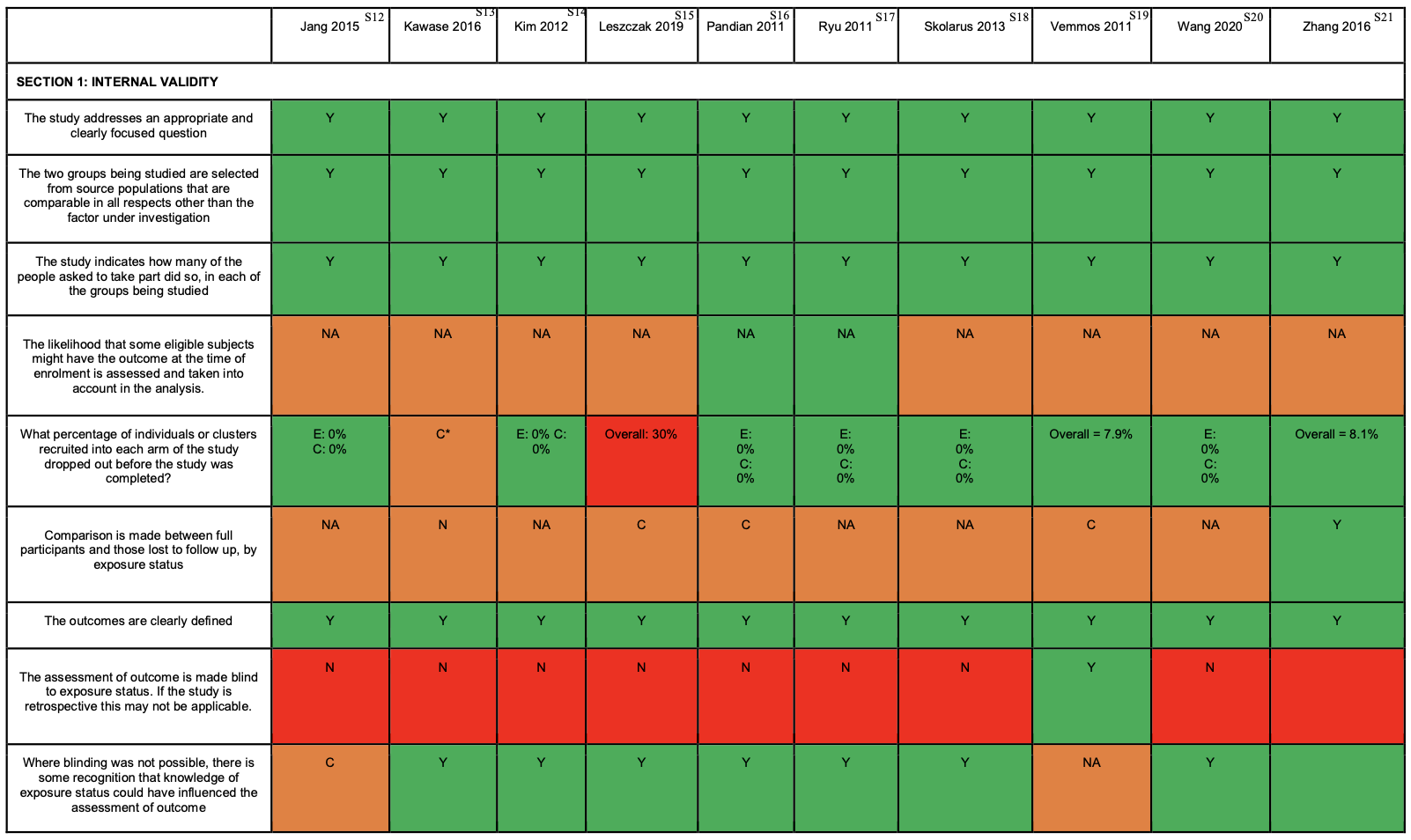

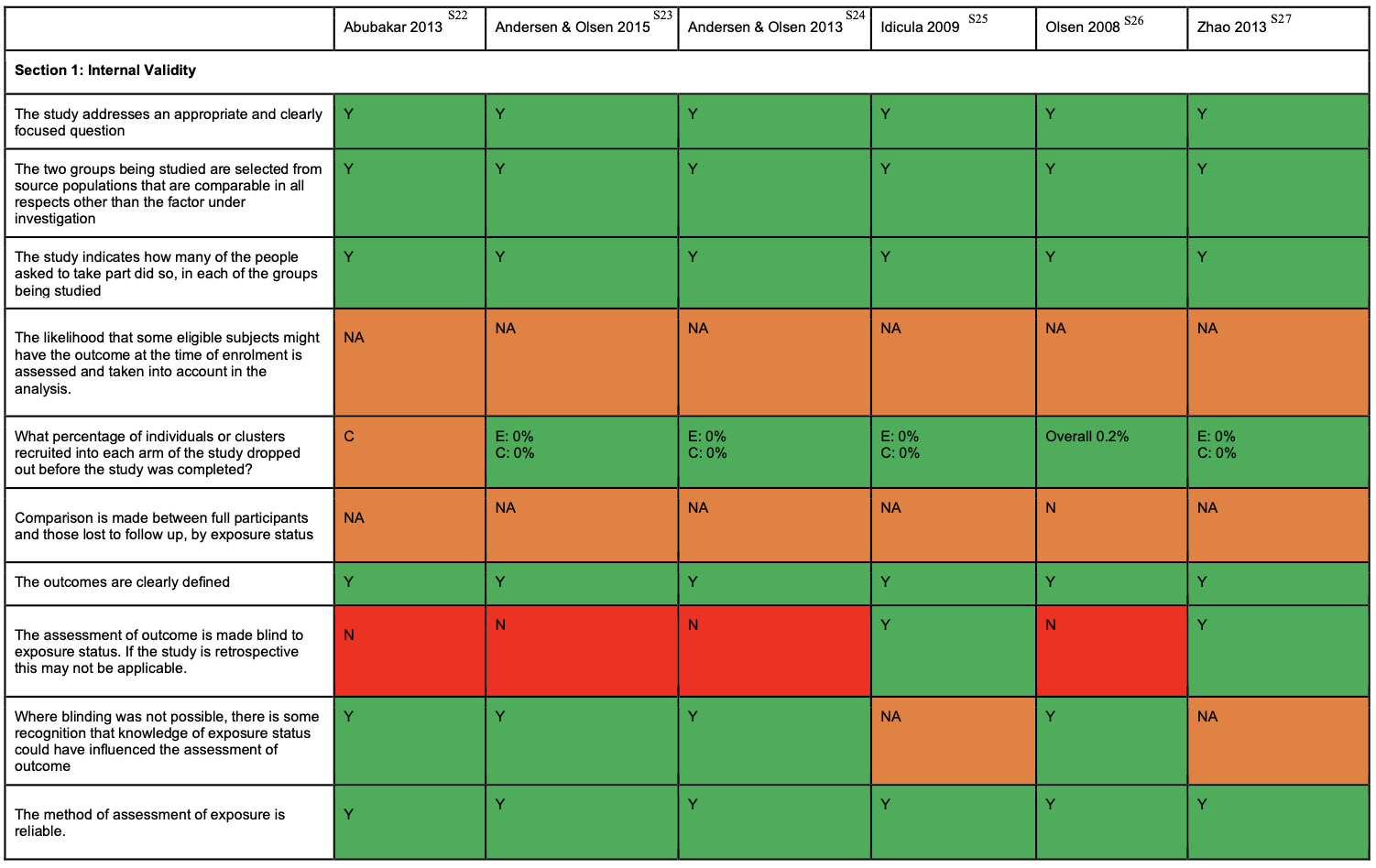


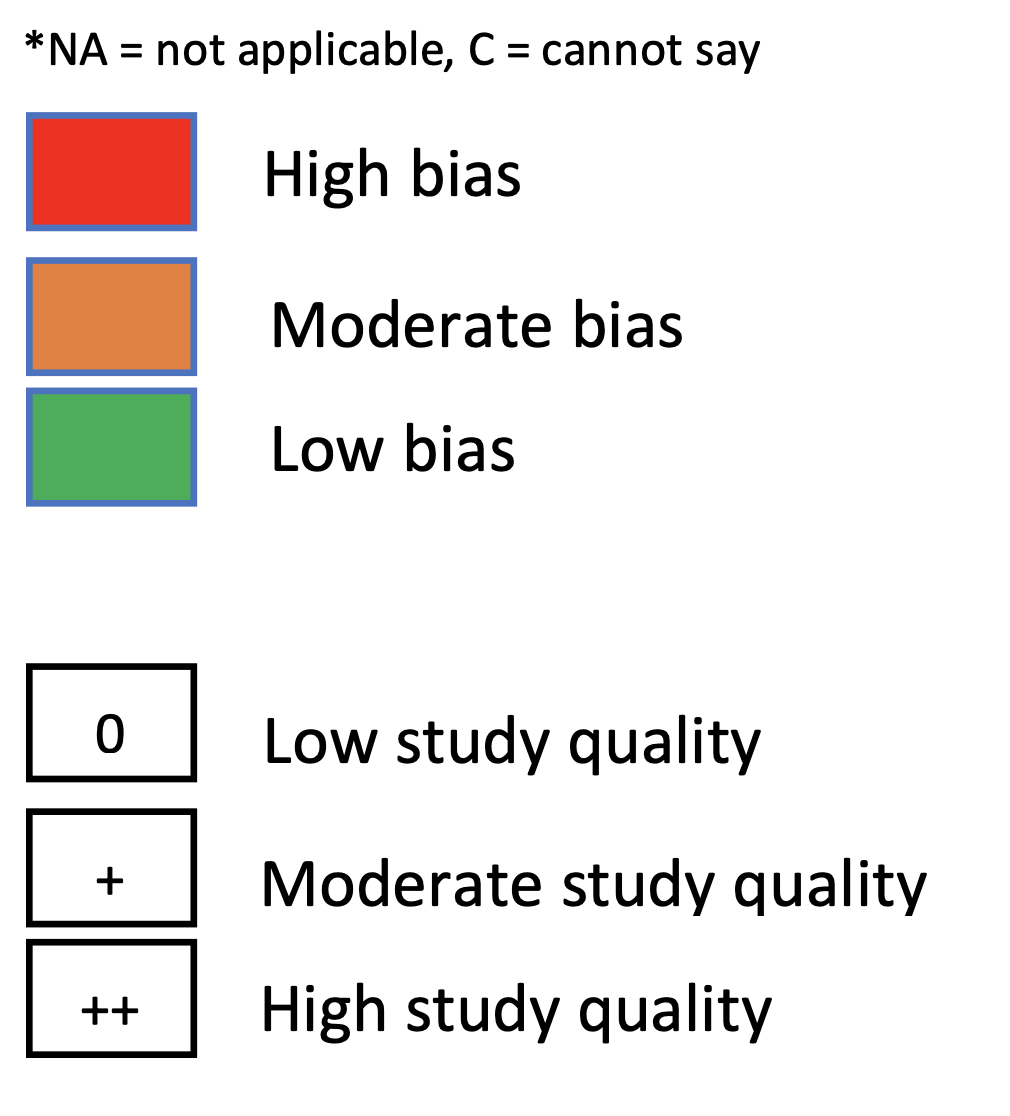


Table I: Critical appraisal using SIGN guidelines of each paper included in the review

S1 - Bell CL, Lacroix A, Masaki K, et al. Prestroke factors associated with poststroke mortality and recovery in older women in the women’s health initiative. Journal of the American Geriatrics Society. 2013;61(8). doi:10.1111/jgs.12361

S2 - Bhalla A, Sankaralingam S, Dundas R, Swaminathan R, Wolfe CDA, Rudd AG. Influence of raised plasma osmolality on clinical outcome after acute stroke. Stroke. 2000;31(9). doi:10.1161/01.STR.31.9.2043

S3 - Branscheidt M, Schneider J, Michel P, et al. No impact of body mass index on outcome in stroke patients treated with IV thrombolysis BMI and IV thrombolysis outcome. PLoS ONE. 2016;11(10). doi:10.1371/journal.pone.0164413

S4 - Carter AM, Catto AJ, Mansfield MW, Bamford JM, Grant PJ. Predictive variables for mortality after acute ischemic stroke. Stroke. 2007;38(6). doi:10.1161/STROKEAHA.106.474569

S5 - Davalos A, Ricart W, Gonzalez-Huix F, et al. Effect of malnutrition after acute stroke on clinical outcome. Stroke. 1996;27(6). doi:10.1161/01.STR.27.6.1028

S6 - Davalos A, Ricart W, Gonzalez-Huix F, et al. Effect of malnutrition after acute stroke on clinical outcome. Stroke. 1996;27(6). doi:10.1161/01.STR.27.6.1028

S7 - Davis JP, Wong AA, Schluter PJ, Henderson RD, O’Sullivan JD, Read SJ. Impact of premorbid undernutrition on outcome in stroke patients. Stroke. 2004;35(8). doi:10.1161/01.STR.0000135227.10451.c9

S8 - FOOD Trial. Poor nutritional status on admission predicts poor outcomes after stroke observational data from the food trial. Stroke. 2003;34(6). doi:10.1161/01.STR.0000074037.49197.8C

S9 - Gomes F, Emery PW, Weekes CE. Risk of Malnutrition Is an Independent Predictor of Mortality, Length of Hospital Stay, and Hospitalization Costs in Stroke Patients. Journal of Stroke and Cerebrovascular Diseases. 2016;25(4). doi:10.1016/j.jstrokecerebrovasdis.2015.12.017

S10 - Gariballa SE, Parker SG, Taub N, Castleden CM. Influence of nutritional status on clinical outcome after acute stroke. American Journal of Clinical Nutrition. 1998;68(2). doi:10.1093/ajcn/68.2.275

S11 - Gariballa SE, Parker SG, Taub N, Castleden M. Nutritional status of hospitalized acute stroke patients. British Journal of Nutrition. 1998;79(6). doi:10.1079/bjn19980085

S12 - Jang SY, Shin Y il, Kim DY, et al. Effect of obesity on functional outcomes at 6 months post-stroke among elderly Koreans: A prospective multicentre study. BMJ Open. 2015;5(12). doi:10.1136/bmjopen-2015-008712

S13 - Kawase S, Kowa H, Suto Y, et al. Association between body mass index and outcome in Japanese ischemic stroke patients. Geriatrics and Gerontology International. 2016;17(3). doi:10.1111/ggi.12713

S14 - Kim BJ, Lee SH, Jung KH, Yu KH, Lee BC, Roh JK. Dynamics of obesity paradox after stroke, related to time from onset, age, and causes of death. Neurology. 2012;79(9). doi:10.1212/WNL.0b013e318266fad1

S15 - Leszczak J, Czenczek-Lewandowska E, Przysada G, et al. Association between body mass index and results of rehabilitation in patients after stroke: A 3-month observational follow-up study. Medical Science Monitor. 2019;25. doi:10.12659/MSM.915586

S16 - Pandian JD, Jyotsna R, Singh R, et al. Premorbid nutrition and short term outcome of stroke: A multicentre study from India. Journal of Neurology, Neurosurgery and Psychiatry. 2011;82(10). doi:10.1136/jnnp.2010.233429

S17 - Ryu WS, Lee SH, Kim CK, Kim BJ, Yoon BW. Body mass index, initial neurological severity and long-term mortality in ischemic stroke. Cerebrovascular Diseases. 2011;32(2). doi:10.1159/000328250

S18 - Skolarus LE, Sanchez BN, Levine DA, et al. Association of body mass index and mortality after acute ischemic stroke. Circulation: Cardiovascular Quality and Outcomes. 2013;7(1). doi:10.1161/CIRCOUTCOMES.113.000129

S19 - Vemmos K, Ntaios G, Spengos K, et al. Association between obesity and mortality after acute first-ever stroke: The obesity-stroke paradox. Stroke. 2011;42(1). doi:10.1161/STROKEAHA.110.593434

S20 - Wang J, Li J, Li M, et al. Association between dynamic obesity and mortality in patients with first-ever ischemic stroke: A hospital-based prospective study. Medicine. 2020;99(38). doi:10.1097/MD.0000000000022243

S21 - Zhang Q, Lei YX, Wang Q, et al. Serum albumin level is associated with the recurrence of acute ischemic stroke. American Journal of Emergency Medicine. 2016;34(9). doi:10.1016/j.ajem.2016.06.049

S22 - Abubakar S, Sabir A, Ndakotsu M, Imam M, Tasiu M. Low admission serum albumin as prognostic determinant of 30-day case fatality and adverse functional outcome following acute ischemic stroke. Pan African Medical Journal. 2013;14. doi:10.11604/pamj.2013.14.53.1941

S23 - Andersen KK, Olsen TS. The Obesity Paradox in Stroke: Lower Mortality and Lower Risk of Readmission for Recurrent Stroke in Obese Stroke Patients. International Journal of Stroke. 2015;10(1). doi:10.1111/ijs.12016

S24 - Andersen KK, Olsen TS. Body Mass Index and Stroke: Overweight and Obesity Less Often Associated with Stroke Recurrence. Journal of Stroke and Cerebrovascular Diseases. 2013;22(8). doi:10.1016/j.jstrokecerebrovasdis.2013.06.031

S25 - Idicula TT. Acute ischemic stroke FacAcute ischemic stroke Factors that predict outcome. BMC Neurology. Published online 2009.

S26 - Olsen TS, Dehlendorff C, Petersen HG, Andersen KK. Body mass index and poststroke mortality. Neuroepidemiology. 2008;30(2). doi:10.1159/000118945

S27 - Zhao L, Du W, Zhao X, et al. Favorable functional recovery in overweight ischemic stroke survivors: Findings from the China National Stroke Registry. Journal of Stroke and Cerebrovascular Diseases. 2014;23(3). doi:10.1016/j.jstrokecerebrovasdis.2013.10.002

Table S2


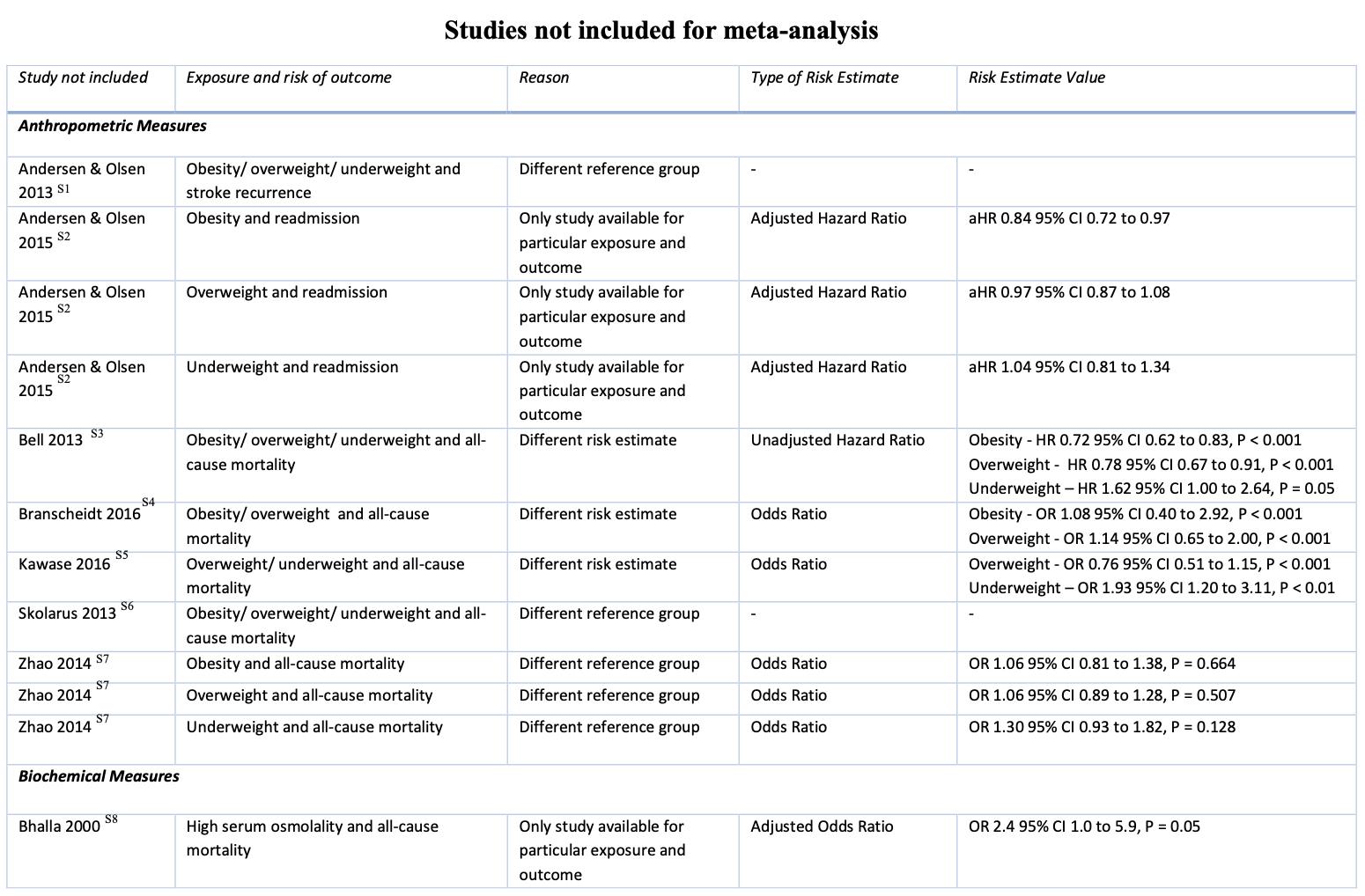


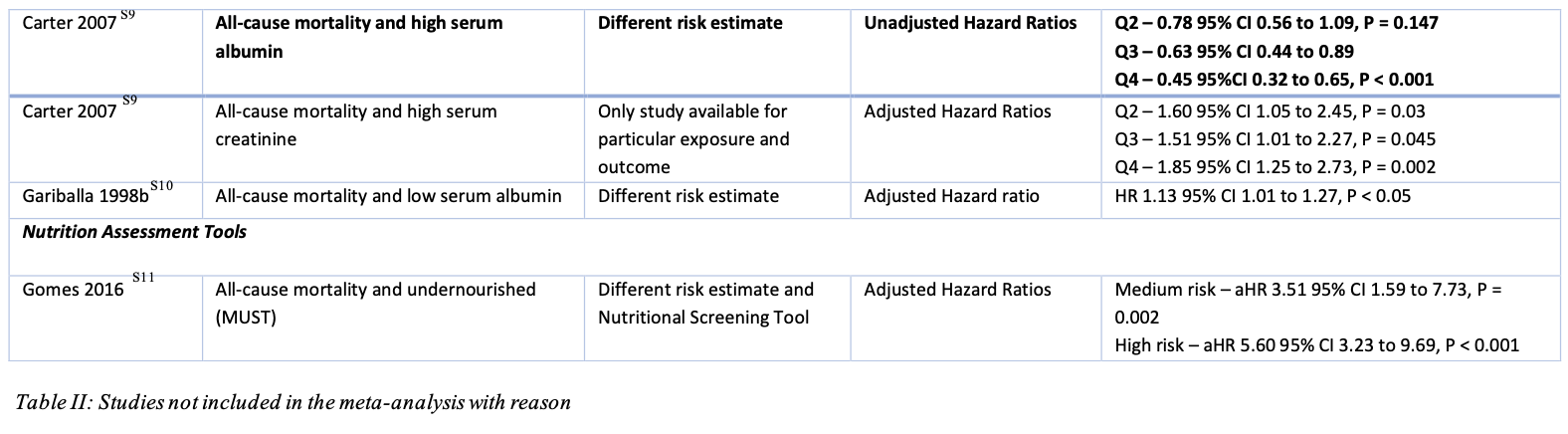


S1 - Andersen KK, Olsen TS. Body Mass Index and Stroke: Overweight and Obesity Less Often Associated with Stroke Recurrence. Journal of Stroke and Cerebrovascular Diseases. 2013;22(8). doi:10.1016/j.jstrokecerebrovasdis.2013.06.031

S2 - Andersen KK, Olsen TS. The Obesity Paradox in Stroke: Lower Mortality and Lower Risk of Readmission for Recurrent Stroke in Obese Stroke Patients. International Journal of Stroke. 2015;10(1). doi:10.1111/ijs.12016

S3 - Bell CL, Lacroix A, Masaki K, et al. Prestroke factors associated with poststroke mortality and recovery in older women in the women’s health initiative. Journal of the American Geriatrics Society. 2013;61(8). doi:10.1111/jgs.12361

S4 - Branscheidt M, Schneider J, Michel P, et al. No impact of body mass index on outcome in stroke patients treated with IV thrombolysis BMI and IV thrombolysis outcome. PLoS ONE. 2016;11(10). doi:10.1371/journal.pone.0164413

S5 - Kawase S, Kowa H, Suto Y, et al. Association between body mass index and outcome in Japanese ischemic stroke patients. Geriatrics and Gerontology International. 2016;17(3). doi:10.1111/ggi.12713

S6 - Skolarus LE, Sanchez BN, Levine DA, et al. Association of body mass index and mortality after acute ischemic stroke. Circulation: Cardiovascular Quality and Outcomes. 2013;7(1). doi:10.1161/CIRCOUTCOMES.113.000129

S7 - Zhao L, Du W, Zhao X, et al. Favorable functional recovery in overweight ischemic stroke survivors: Findings from the China National Stroke Registry. Journal of Stroke and Cerebrovascular Diseases. 2014;23(3). doi:10.1016/j.jstrokecerebrovasdis.2013.10.002

S8 - Bhalla A, Sankaralingam S, Dundas R, Swaminathan R, Wolfe CDA, Rudd AG. Influence of raised plasma osmolality on clinical outcome after acute stroke. Stroke. 2000;31(9). doi:10.1161/01.STR.31.9.2043

S9 - Carter AM, Catto AJ, Mansfield MW, Bamford JM, Grant PJ. Predictive variables for mortality after acute ischemic stroke. Stroke. 2007;38(6). doi:10.1161/STROKEAHA.106.474569

S10 - Gariballa SE, Parker SG, Taub N, Castleden M. Nutritional status of hospitalized acute stroke patients. British Journal of Nutrition. 1998;79(6). doi:10.1079/bjn19980085

S11 - Gomes F, Emery PW, Weekes CE. Risk of Malnutrition Is an Independent Predictor of Mortality, Length of Hospital Stay, and Hospitalization Costs in Stroke Patients. Journal of Stroke and Cerebrovascular Diseases. 2016;25(4). doi:10.1016/j.jstrokecerebrovasdis.2015.12.017


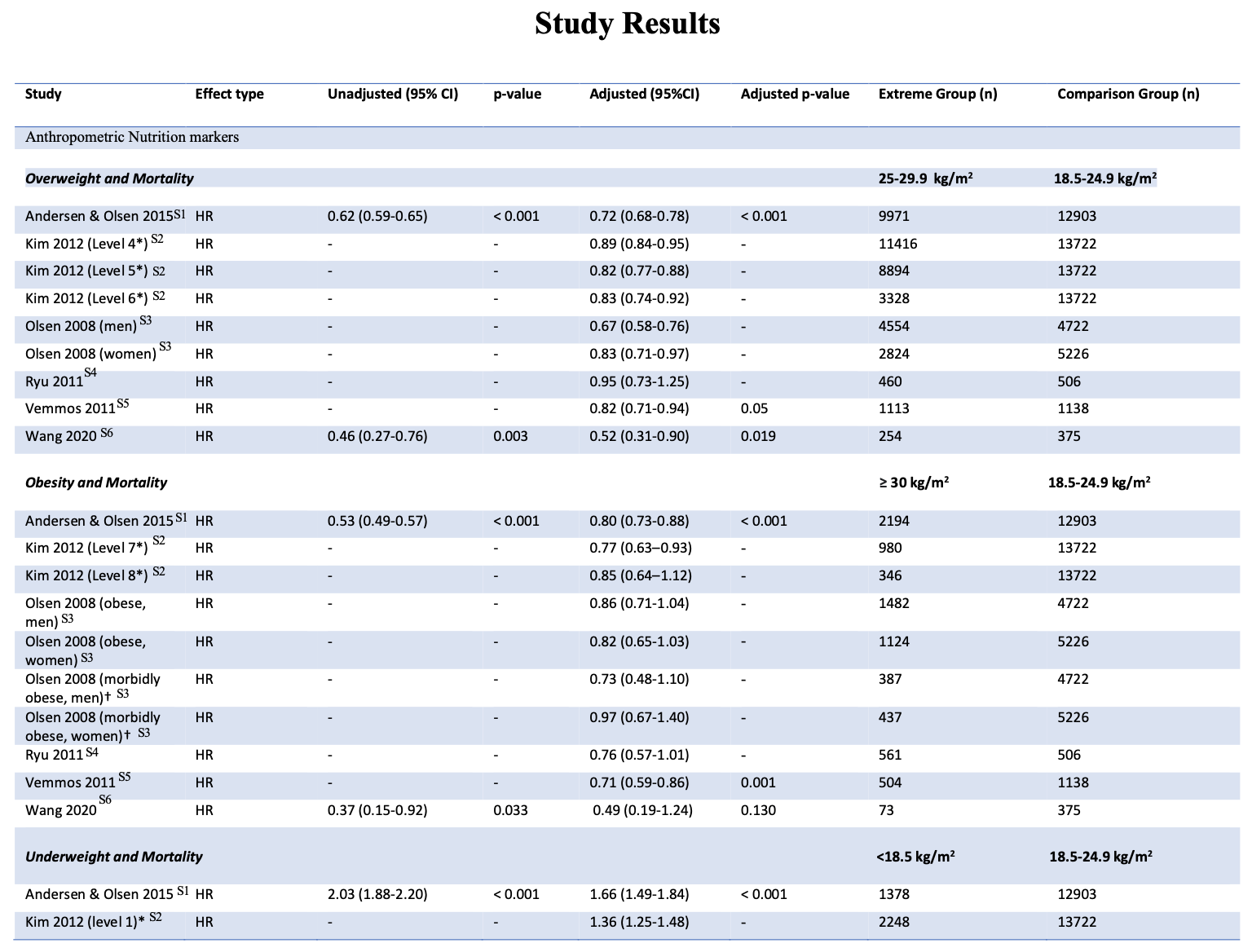
Table S3


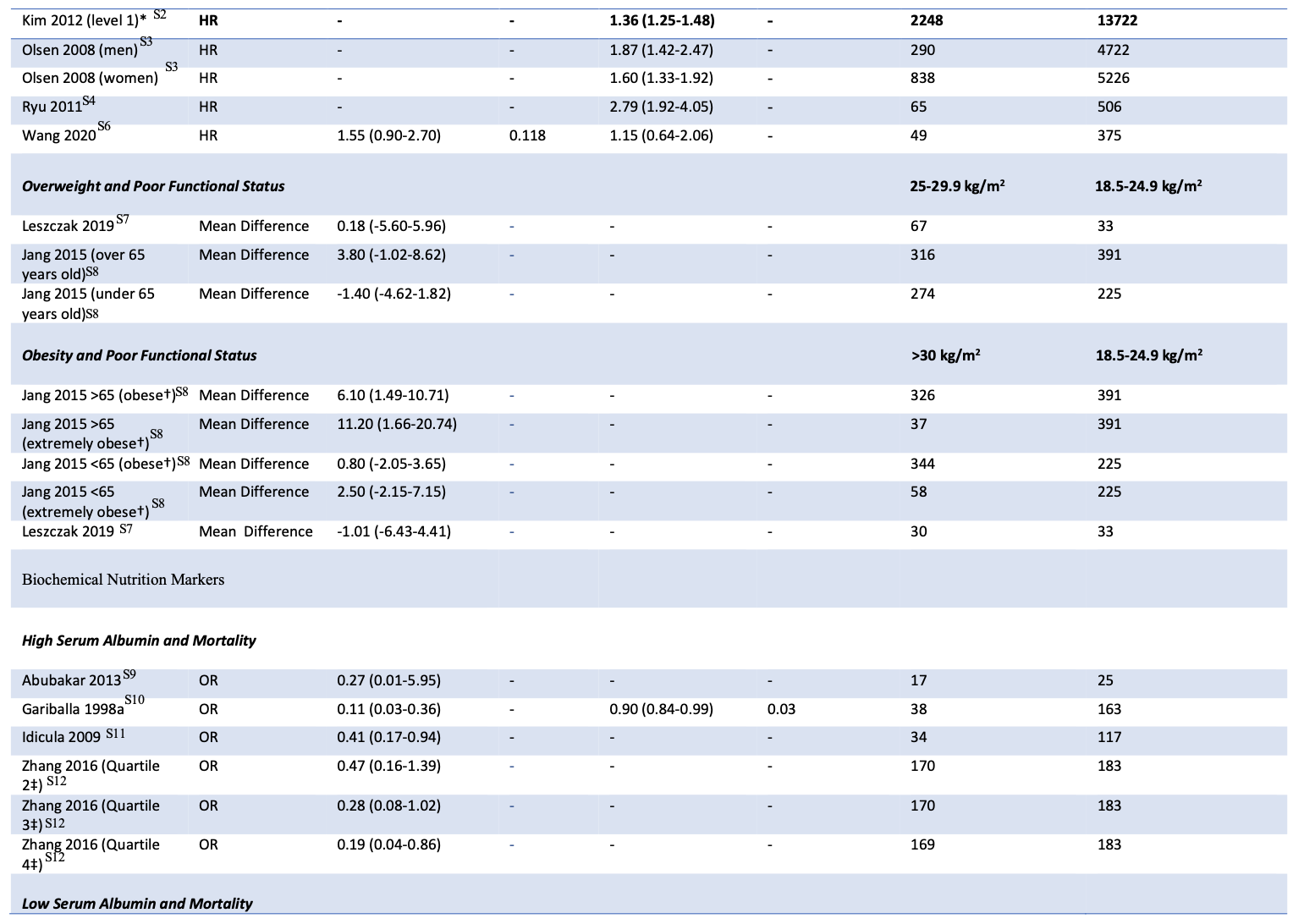


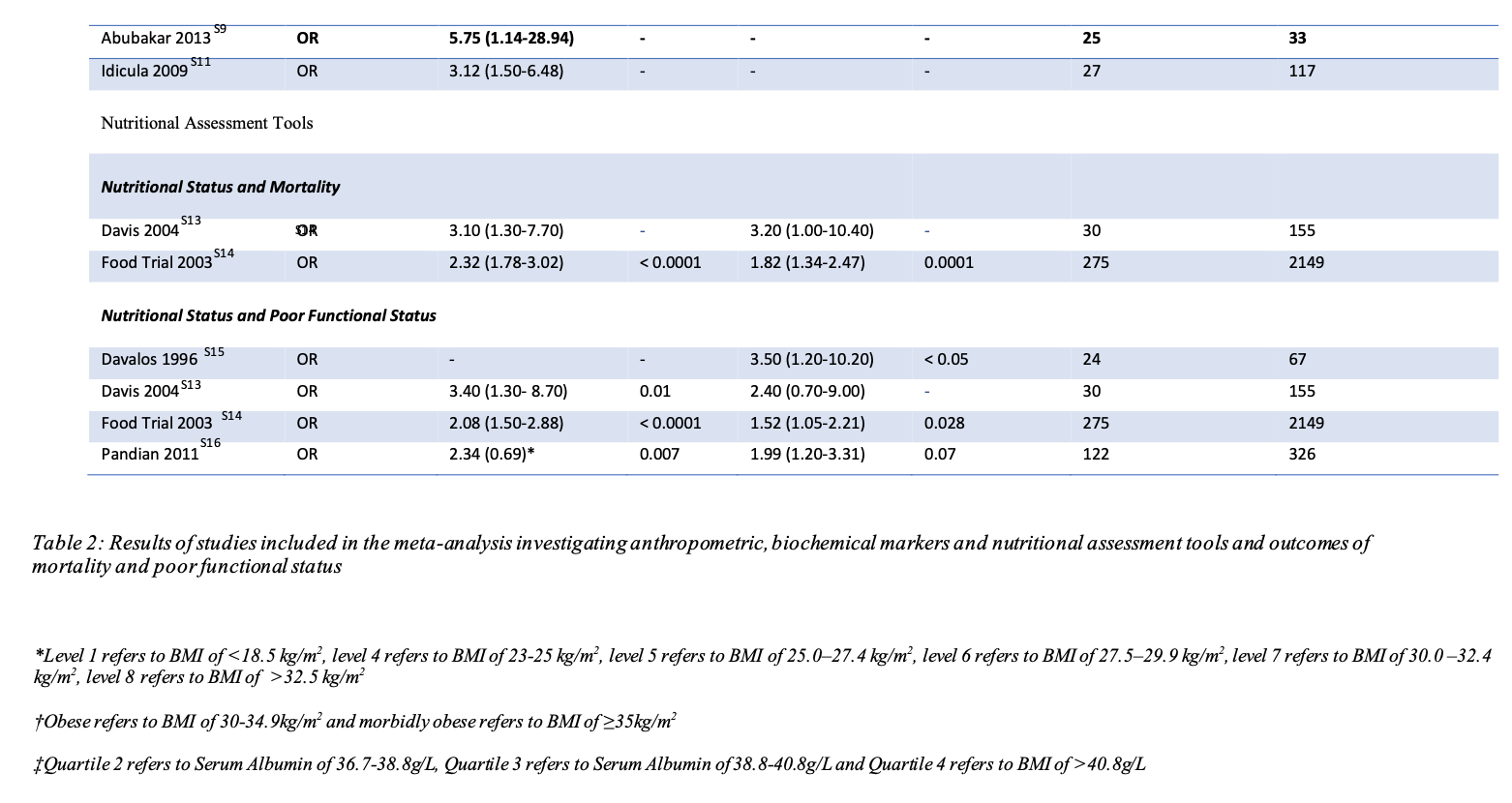


S1 - Andersen KK, Olsen TS. The Obesity Paradox in Stroke: Lower Mortality and Lower Risk of Readmission for Recurrent Stroke in Obese Stroke Patients. International Journal of Stroke. 2015;10(1). doi:10.1111/ijs.12016

S2 - Kim BJ, Lee SH, Jung KH, Yu KH, Lee BC, Roh JK. Dynamics of obesity paradox after stroke, related to time from onset, age, and causes of death. Neurology. 2012;79(9). doi:10.1212/WNL.0b013e318266fad1

S3 - Olsen TS, Dehlendorff C, Petersen HG, Andersen KK. Body mass index and poststroke mortality. Neuroepidemiology. 2008;30(2). doi:10.1159/000118945

S4 - Ryu WS, Lee SH, Kim CK, Kim BJ, Yoon BW. Body mass index, initial neurological severity and long-term mortality in ischemic stroke. Cerebrovascular Diseases. 2011;32(2). doi:10.1159/000328250

S5 - Vemmos K, Ntaios G, Spengos K, et al. Association between obesity and mortality after acute first-ever stroke: The obesity-stroke paradox. Stroke. 2011;42(1). doi:10.1161/STROKEAHA.110.593434

S6 - Wang J, Li J, Li M, et al. Association between dynamic obesity and mortality in patients with first-ever ischemic stroke: A hospital-based prospective study. Medicine. 2020;99(38). doi:10.1097/MD.0000000000022243

S7 - Leszczak J, Czenczek-Lewandowska E, Przysada G, et al. Association between body mass index and results of rehabilitation in patients after stroke: A 3-month observational follow-up study. Medical Science Monitor. 2019;25. doi:10.12659/MSM.915586

S8 - Jang SY, Shin Y il, Kim DY, et al. Effect of obesity on functional outcomes at 6 months post-stroke among elderly Koreans: A prospective multicentre study. BMJ Open. 2015;5(12). doi:10.1136/bmjopen-2015-008712

S9 - Abubakar S, Sabir A, Ndakotsu M, Imam M, Tasiu M. Low admission serum albumin as prognostic determinant of 30-day case fatality and adverse functional outcome following acute ischemic stroke. Pan African Medical Journal. 2013;14. doi:10.11604/pamj.2013.14.53.1941

S10 - Gariballa SE, Parker SG, Taub N, Castleden CM. Influence of nutritional status on clinical outcome after acute stroke. American Journal of Clinical Nutrition. 1998;68(2). doi:10.1093/ajcn/68.2.275

S11 - Idicula TT. Acute ischemic stroke FacAcute ischemic stroke Factors that predict outcome. BMC Neurology. Published online 2009.

S12 - Zhang Q, Lei YX, Wang Q, et al. Serum albumin level is associated with the recurrence of acute ischemic stroke. American Journal of Emergency Medicine. 2016;34(9). doi:10.1016/j.ajem.2016.06.049

S13 - Davis JP, Wong AA, Schluter PJ, Henderson RD, O’Sullivan JD, Read SJ. Impact of premorbid undernutrition on outcome in stroke patients. Stroke. 2004;35(8). doi:10.1161/01.STR.0000135227.10451.c9

S14 - FOOD Trial. Poor nutritional status on admission predicts poor outcomes after stroke observational data from the food trial. Stroke. 2003;34(6). doi:10.1161/01.STR.0000074037.49197.8C

S15 - Davalos A, Ricart W, Gonzalez-Huix F, et al. Effect of malnutrition after acute stroke on clinical outcome. Stroke. 1996;27(6). doi:10.1161/01.STR.27.6.1028

S16 - Pandian JD, Jyotsna R, Singh R, et al. Premorbid nutrition and short term outcome of stroke: A multicentre study from India. Journal of Neurology, Neurosurgery and Psychiatry. 2011;82(10). doi:10.1136/jnnp.2010.233429

Table S4

Table IV: Full PRSMA Checklist for this Review
